# The ufmylation modification of ribosomal protein L10 in the development of pancreatic adenocarcinoma

**DOI:** 10.1038/s41419-023-05877-y

**Published:** 2023-06-07

**Authors:** Kun Wang, Siyu Chen, Yue Wu, Yang Wang, Yousheng Lu, Yanzi Sun, Yijun Chen

**Affiliations:** 1 State Key Laboratory of Natural Medicines and Laboratory of Chemical Biology, China Pharmaceutical University, 639 Longmian Ave., Nanjing, Jiangsu Province 211198 China; 2 Jiangsu Cancer Hospital & Jiangsu Institute of Cancer Research & the Affiliated Cancer Hospital of Nanjing Medical University, 42 Baiziting, Kunlun Road, Nanjing, Jiangsu Province 210009 China; 3 Chongqing Innovation Institute of China Pharmaceutical University, Chongqing, 401135 China

**Keywords:** Ubiquitylated proteins, Cancer, Stem cells

## Abstract

Pancreatic adenocarcinoma (PAAD) is the most malignant cancer with a high mortality rate. Despite the association of ribosomal protein L10 (RPL10) with PAAD and previous reports on RPL26 ufmylation, the relationship between RPL10 ufmylation and PAAD development remains unexplored. Here, we report the dissection of ufmylating process of RPL10 and potential roles of RPL10 ufmylation in PAAD development. The ufmylation of RPL10 was confirmed in both pancreatic patient tissues and cell lines, and specific modification sites were identified and verified. Phenotypically, RPL10 ufmylation significantly increased cell proliferation and stemness, which is principally resulted from higher expression of transcription factor KLF4. Moreover, the mutagenesis of ufmylation sites in RPL10 further demonstrated the connection of RPL10 ufmylation with cell proliferation and stemness. Collectively, this study reveals that PRL10 ufmylation plays an important role to enhance the stemness of pancreatic cancer cells for PAAD development.

## Introduction

Pancreatic adenocarcinoma (PAAD) is a solid malignancy with the least treatment options, resulting in only 9% of five-year survival rate [1, 2]. Because most patients are found in the advanced stages, they automatically miss the best chance for treatments. Although surgery and adjuvant chemotherapy are commonly used for treating PAAD patients, the treatments usually do not produce desired therapeutic effects [3]. Gemcitabine, a standard therapy for locally advanced and metastatic PAAD, cannot provide satisfactory benefits to PAAD patients, developing obvious chemotherapy resistance within weeks [4]. Meanwhile, combination therapy with gemcitabine also shows limited improvement compared to gemcitabine alone [5]. Therefore, novel therapeutic strategies for PAAD are urgently needed for improving the outcomes.

Similar to ubiquitination, ufmylation system is consisted of conjugating substrate ubiquitin-like modifier 1 (UFM1) and three enzymes, including ubiquitin-like modifier 1 activating enzyme 5 (UBA5, E1), UFM1 conjugating enzyme 1 (UFC1, E2) and UFM1-protein ligase 1 (UFL1, E3) [6]. Additionally, the formation of E3 ligase complex with adaptor proteins for ufmylation is required as a non-canonical mode [7]. Reversibly, the conjugation of UFM1 on protein substrates at lysine residues can be cleaved by UFM1-specific protease 2 (UFSP2) to regulate the ufmylation in human cells and tissues [8, 9]. Recently, UFSP1, possessing different substrate specificity from UFSP2, is revealed to promote UFM1 maturation for ufmylation [10]. Previous studies have linked ufmylation with various cellular functions, particularly tumorigenesis. The ufmylation of tumor suppressor p53 can increase its stability through antagonizing the ubiquitination-mediated degradation [11]. In the presence of 17-estradiol, the ufmylation of activating signal cointegrator 1 promotes the development of breast cancer [12]. In the case of PAAD, a small molecule DKM 2-93 can covalently modify the catalytic cysteine residue of UBA5 to prevent the growth of pancreatic cancer cells for tumorigenesis [13]. Thus, the removal of ufmylation modifications by inhibiting UBA5 contributes to the slowdown of PAAD development [13]. However, the functional involvement of ufmylation system, the protein substrates to be ufmylated, the regulation of ufmylation process and the correlation between ufmylation and PAAD development are less understood.

Ribosomal proteins are a class of accessory proteins essential for the ribosome assembly and protein synthesis [14]. Additionally, increasing evidences indicate that this class of proteins exhibit extra-ribosomal functions. Ribosomal protein L26 (RPL26) is ufmylated for the translocation-associated quality control of protein homeostasis in endoplasmic reticulum [15, 16]. Meanwhile, as a component of 60S subunit and a member of ribosomal proteins, ribosomal protein L10 (RPL10) was first reported in Wilms’ tumor cells [17]. In addition to the involvement in the assembly and maturation of 60S subunit by RPL10 [18, 19], this ribosomal protein shows a variety of extra-ribosomal functions [20]. Regarding the relationship with cancer, RPL10 has been implicated as a biomarker and target for human epithelial ovarian cancer [21, 22]. In prostate cancer, the expression of RPL10 is gradually increased with the progress of the disease [23–25]. Moreover, 9.8% children in the population with acute T-lymphoblastic leukemia are found to have a R98S mutation in RPL10, driving IRES-dependent BCL-2 translation and amplifying JAK-STAT signaling [26–28]. Collectively, RPL10, especially its extra-ribosomal functions, has been suggested to associate with different tumorigeneses.

Previously, using dimethyl-amino-parthenolide (DMAPT) as a molecular probe, we found that DMAPT can directly bind RPL10 and synergistically exhibit its anti-proliferative activity from the inhibition of p65 in pancreatic cancer cells [29]. Additionally, RPL10 functioned as a buffering molecule to balance and regulate ROS level in mitochondrion of pancreatic cancer cells [30]. Despite the absence of post-translational modifications in the structure of eukaryotic 60 S subunit complexed with RPL10 and other ribosomal proteins including RPL26 by cryo-electron microscopy [31, 32], the ufmylation of RPL10 was found in E14 embryonic stem cells through a proteomics analysis of ribosome-associating proteins [33]. This strongly suggests that RPL10 ufmylation may play an important regulatory role in human cells. Although actual functions of RPL10 ufmylation remain unknown, we speculate that this unprecedented modification could be associated with PAAD development based on our previous findings [34, 35].

In this study, the ufmylation of RPL10 was confirmed in the tissues of PAAD patients and pancreatic cancer cell lines, and this modification was mediated by UFL1 at specific sites and cleaved by UFSP2. Additionally, the decrease of RPL10 ufmylation inhibited pancreatic cancer cell proliferation and stemness. Furthermore, the degree between RPL10 ufmylation and cell stemness was positively regulated by the transcription factor KLF4. Moreover, RPL10 ufmylation was clearly correlated with the tumorigenesis through the increase of cell surface markers for stemness and KLF4 expression. Finally, mutagenesis of the specific sites of ufmylation in RPL10 impeded the proliferation and stemness of pancreatic cancer cells. Taken together, these findings have laid a foundation for comprehensive elucidation of functional roles of RPL10 ufmylation in tumorigenesis and provided a potential therapeutic opportunity for PAAD.

## Materials and methods

### Cell lines and cell cultures

The pancreatic cancer cell lines of PANC-1 and Mia PaCa-2 were bought from KeyGEN Biotech Co., Ltd (Jiangsu, China) and cultured with Dulbecco’s modified eagle medium (DMEM, Gibco, California, USA) containing 10% fetal bovine serum (FBS, Biological Industries, Beit-Haemek, Israel) at 37 °C in incubator with 5% CO_2_. All human cell lines have been authenticated using STR profiling, and mycoplasma contamination was detected to be free. The cells at 80–90% confluence were detached with digestion solution (Gibco, California, USA) and washed with phosphate buffer saline (PBS, Gibco, California, USA).

### Clinical specimens

The tumor tissues and adjacent normal tissues from five PAAD patients were freshly frozen in Jiangsu Cancer Hospital (Nanjing, China), and the patient samples were examined by pathologists for confirming the diagnosis.

### Plasmid construction and transfection, lentiviral packaging and selection of stable cells

RNA interference segment of UFL1 (Accession: NM_015323.5) and RPL10 (Accession: CR542069.1) (Table S1) was constructed into GV248 plasmid (Gene Chem, Shanghai, China). The GV248-NC, GV248-UFL1i or GV248-RPL10i was transfected into HEK293T cells respectively with a packaging plasmid mixture to produce lentivirus. All lentiviruses were incubated with PANC-1 and Mia PaCa-2 cells for the transfection after starvation without FBS for 4 h, and then the supernatant was aspirated and replaced by fresh culture medium after 12 h incubation. The cells transfected by lentiviruses were filtered out with puromycin for subsequent studies.

The coding sequence of UFSP2 (Accession: BC010493.1) was amplified from cDNA in PANC-1 cells by standard Polymerase Chain Reaction (PCR) and ligated into pcDNA3.1 plasmid for the construction of UFSP2 overexpression vector. The pcDNA3.1-UFSP2 was transfected into PANC-1 and Mia PaCa-2 cells for overexpressing UFSP2.

Each mutant sequence of RPL10 was generated with Gene Mutation Reagent Kit (Beyotime, Shanghai, China) and ligated into pLenti-GII-CMV-CBH-GFP-2A-Puro-Amp plasmid (ABM, Jiangsu, China). These plasmids were transfected into HEK293T cells with a packaging plasmid mixture to produce lentivirus. All lentiviruses were transfected into PANC-1 cells after starvation without FBS for 4 h, and then the supernatant was aspirated and replaced by fresh culture medium after 12 h incubation. The cells transfected by lentiviruses were filtered out with puromycin for subsequent studies.

### Animals

Four-week old female NPG mice were purchased from Beijing Vital River Laboratory Animal Technology Co., Ltd (Jiangsu, China). The mice had a free access to laboratory chow and water with constant conditions (23 ± 2 °C, LD 12:12) followed by a week acclimatization.

### Ethics statement

All animal welfares and ethics as well as experimental procedures were approved and carried out in compliance with the ethical review committee of China Pharmaceutical University (Approval no. 2021-06-001). All studies carried out on human specimens were approved by the ethical review committee of Jiangsu Cancer Hospital (Approval no. 20200312), conducting in compliance with the ethical guidelines. A consent for each patient was informed.

### Generation and treatments of xenografted mice with stable cells after knockdown of ufl1

Sixteen NPG mice were randomly assigned to two groups of NC and UFL-KD (ufl1 knockdown). Tumor xenografts with pancreatic cancer cells were established by subcutaneously injecting the suspension (1 × 10^7^ cells) of PANC-1 cells with stable gene knockdown into the right flank region of the mice. No blinding method was used for the injection. The volumes of subcutaneous tumors were determined twice a week and calculated via the following formula: V = d_max_×d_min_^2^/2, where V represents the tumor volume, d_max_ is the maximum perpendicular diameter and d_min_ is the minimum perpendicular diameter. The mice were sacrificed after 49 days, and tumor tissues were isolated, weighed and frozen in liquid nitrogen for subsequent analyses.

### Co-Immunoprecipitation

Cells or isolated tumor tissues were lysed in NP-40 (EDTA 0.5 mM, Tris 50 mM, NaCl 150 mM, NP-40 1%, MgCl_2_ 0.5 mM, pH 7.4) lysis buffer (Sigma, San Francisco, USA) with protease inhibitors (Thermo Fisher, Massachusetts, USA) for 10 min at 4 °C. After centrifugation at 12,000 x *g* for 10 min at 4 °C, the supernatants were collected, and the concentration of protein in each lysate was quantitated by BCA assay kit (Beyotime, Shanghai, China). The lysates were incubated with anti-RPL10 antibody (Santa Cruz, #H2409), anti-UFM1 antibody (Abcam, #ab109305), anti-RPS3 antibody (CST technology, #9538) or anti-flag antibody (CST technology, #8146) for 16 h at 4 °C. Then, 30 μl Agarose (Millipore, Massachusetts, USA) was washed by PBS and incubated with the mixture of lysate and antibody for additional 1 h at room temperature. Finally, the precipitants were laved with lysis buffer for five times, and mixed with loading buffer to boil for 5 min for subsequent Western blotting.

### Western blot analysis

Lysates from cells or tissues and precipitates from Co-Immunoprecipitation (Co-IP) were analyzed by SDS–PAGE and immunoblotting with following primary antibodies: anti-RPL10 (Thermo Fisher, #MA5-26901), anti-UFM1 (Abcam, #ab109305), anti-UFL1 (Novus, #NBP1-79039), anti-UFSP2 (Abcam, #ab185965) anti-KLF4 (Abcam, #ab215036), anti-Nanog (CST technology, #8822), anti-Oct4 (CST technology, #2750), anti-Sox2 (CST technology, #3579) and anti-flag (CST technology, #8146). Protein bands were stained by enhanced chemiluminescent (ECL, Millipore, Massachusetts, USA) and analyzed by Tanon ChemImaging Systems (Tanon, Shanghai, China).

### Cell proliferation assay

Cells were digested and incubated in 96-well plate for 24 h. The cell proliferation rate was assessed with CCK-8 assay (Dojindo, Kumamoto, Japan) following the manufacturer’s instructions.

### Colony-forming assay

To test the capacity of cells on forming new colonies, 1100 pancreatic cancer cells were digested and incubated in a 6-well plate for 15 days, and cells were washed and dyed with crystal violet. Colonies from the cells were captured and counted. Colony forming rate = (clones / inoculated cells) × 100%.

### Sphere-forming assay

Cells were incubated in 12-well plate of Ultra-low Attachment (Coning, New York, USA) at a density of 1000 cells per well supplemented with DMEM-F12 medium containing epidermal growth factor and fibroblast growth factor. After 10 days, spheres in the plate were photographed and counted under microscope.

### Flow cytometry

Cultured cells were resuspended by trypsinization and incubated with anti-CD24 antibody (BD Biosciences, #56092), anti-ALDH1A1 antibody (CST technology, #65583) or anti-CD44 antibody (BD Biosciences, #560890) for 30 min in ice-bath and washed with PBS under dark condition. Flow cytometry was employed to count cell numbers with fluorescence signals, and the data were analyzed by the software of FlowJo version 10.6.1.

### Immunohistochemistry

Isolated tumor tissues were immediately soaked in paraformaldehyde for fixation and in paraffin for embedment. Then, tissue sections with 4 μm thickness were sliced and processed for immunohistochemical analyses. Immunohistochemical analyses were performed according to the protocol of immunohistochemistry kit (Bioss, Beijing, China) with primary antibodies: anti-Ki67 (Abcam, #ab92742), anti-ALDH1 (Abcam, #ab52492), anti-CD44 (Abcam, #ab51037) and anti-CD24 (Abcam, #ab199140). All sections were photographed by microscope.

### Statistical analysis

No statistical method was used to predetermine the sample size, and no data were excluded from the analysis. Data were expressed as mean ± SD. Tumor volume data were analyzed by two-way ANOVA using GraphPad Prism 8 software, and other data were analyzed by one-way ANOVA or *t*-test using SPSS and plotted using GraphPad Prism software. The variance was similar between the groups that were being statistically compared. A value of *p* < 0.05 denoted statistically significant; a value of *p* < 0.01 denoted statistically very significant.

## Results

### RPL10 ufmylation was correlated with PAAD development

**Fig. 1:**
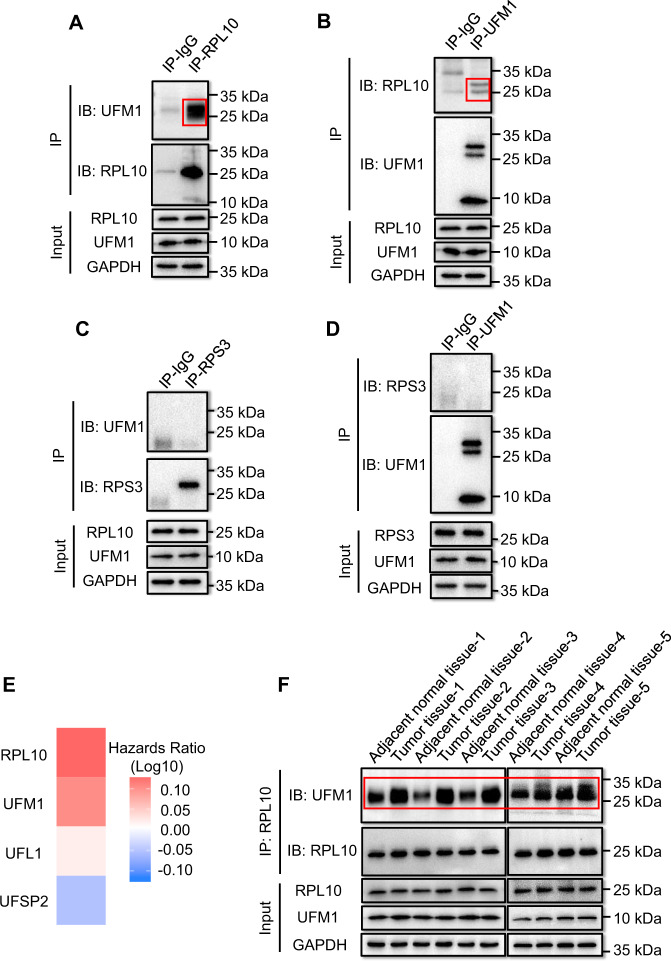
The relationship between RPL10 ufmylation and the development of PAAD. **A** The ufmylation of RPL10 by Co-IP and immunoblotting using anti-RPL10 antibody in PANC-1 cells. **B** The ufmylation of RPL10 by Co-IP and Western blot using anti-UFM1 antibody in PANC-1 cells. **C** The ufmylation of RPS3 by Co-IP and immunoblotting using anti-RPL10 antibody in PANC-1 cells. **D** The ufmylation of RPS3 by Co-IP and Western blot using anti-UFM1 antibody in PANC-1 cells. **E** The hazard ratio of RPL10 and ufmylation related gene expression in PAAD patients (http://gepia2.cancer-pku.cn/#index). The hazards ratios were analyzed by Cox proportional hazard model based on the expressions of the genes in PAAD patient tissues. **F** Comparison of RPL10 ufmylation level between tumor tissues and adjacent normal tissues of PAAD patients (*n* = 5). All experiments were independently repeated for 3 times.

To examine whether RPL10 is modified by UFM1, PANC-1 cells were lysed and detected by Co-IP with antibodies against RPL10 or UFM1, respectively. RPL10 was indeed modified by UFM1 to show ufmylated RPL10 around 25 kDa by Western Blotting (Fig. 1A, B, Supplemental Fig. S3A and Supplemental Fig. S3B). By contrast, ribosomal protein S3 (RPS3), an ufmylated protein reported previously [33], was unable to be modified by UFM1 (Fig. 1C, D, Supplemental Fig. S3C and Supplemental Fig. S3D). These results confirmed the specific conjugation of UFM1 on RPL10 in PANC-1 cells.

To explore whether RPL10 ufmylation is associated with PAAD development, overall survival map was obtained by Cox proportional hazard model based on a multi-gene signature from the examination of the correlation between RPL10 and ufmylation related genes in PAAD patient tissues (http://gepia2.cancer-pku.cn/#index). Obviously, RPL10 showed a positive correlation with modifier UFM1 and E3 ligase UFL1 and a negative correlation with UFSP2 in PAAD patients (Fig. 1E). To experimentally verify such correlations, five pairs of clinical specimens from PAAD patients were used for Co-IP to compare the difference on RPL10 ufmylation between tumor tissues and adjacent normal tissues. As a result, the level of RPL10 ufmylation in PAAD tumor tissues was 2-3 fold higher than that in adjacent normal tissues (Fig. 1F, Supplemental Fig. S3E and Fig. S3F), indicating the close association of RPL10 ufmylation and PAAD development.

### UFM1 was ligated to RPL10 by UFL1 and cleaved by UFSP2

**Fig. 2:**
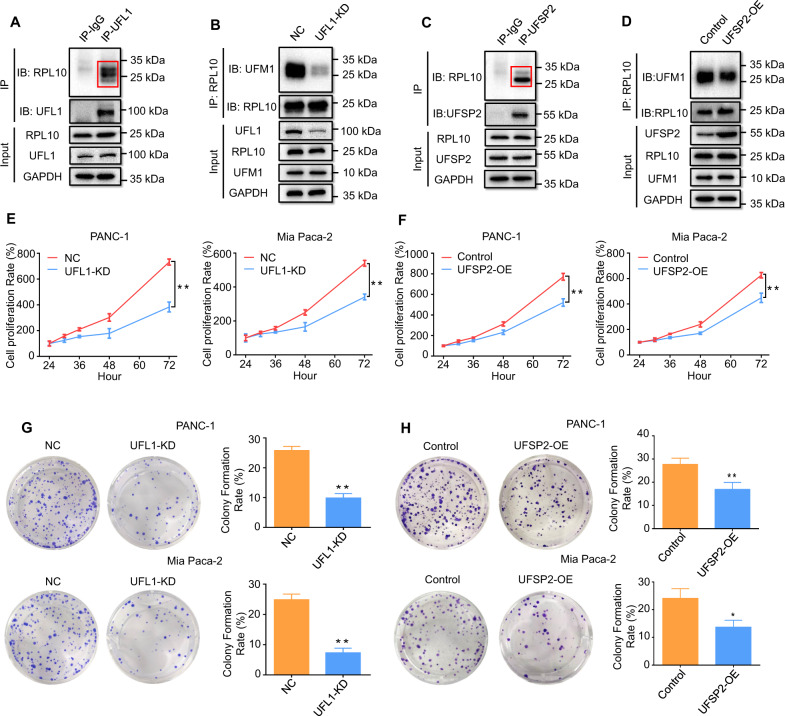
Confirmation of the involvement of UFL1 and UFSP2 in RPL10 ufmylation and the effect on pancreatic cancer cells. **A** Direct binding of RPL10 and UFL1 in PANC-1 cells was detected by Co-IP. **B** The change of RPL10 ufmylation was detected by after UFL1 silencing in PANC-1 cells. **C** Direct binding of UFSP2 and RPL10 in PANC-1 cells by Co-IP. **D** The change of RPL10 ufmylation after overexpressing UFSP2 in PANC-1 cells. **E** The proliferations of PANC-1 cells and Mia PaCa-2 cells after silencing UFL1. The proliferations were detected by CCK-8 method (*n* = 10). **F** The proliferations of PANC-1 and Mia PaCa-2 cells after overexpressing UFSP2. The proliferation rates were detected by CCK-8 method (*n* = 10). **G** The colony formation of PANC-1 and Mia PaCa-2 cells after silencing UFL1. The colonies were stained with crystal violet and analyzed (*n* = 3). **H** The colony formation of PANC-1 and Mia PaCa-2 cells after overexpressing UFSP2. The colonies were stained with crystal violet and analyzed (*n* = 3). All experiments were independently repeated for 3 times, and the data are expressed as mean ± SD. ^*^P < 0.05 indicates statistically significant versus negative control. ^**^P < 0.01 indicates statistically very significant versus negative control.

To ensure whether E3 ligase UFL1 mediates the ufmylation of RPL10, PANC-1 cell lysates were used for Co-IP with anti-UFL1 antibody. Consequently, RPL10 was pulled down by UFL1 (Fig. 2A and Supplemental Fig. S3G). Additionally, when UFL1 was knocked down, the level of RPL10 ufmylation was remarkably reduced (Fig. 2B and Supplemental Fig. S3H).

To determine whether RPL10 is ufmylated in the presence of UFM1, UBA5, UFC1 and UFL1, we incubated the recombinant His-SUMO-RPL10 (34-214) protein, UFM1, UBA5, UFC1 and UFL1 with or without ATP, and analyzed the reaction products by Western blotting using anti-RPL10 antibody. As shown in Supplemental Fig. S1, when all components were present, multiple bands of ufmylated RPL10 showed up starting with the molecular weight of His-SUMO-RPL10 (34-214), ensuring that RPL10 ufmylation is mediated by UFL1.

To clarify whether UFSP2 is responsible for the cleavage of UFM1 from ufmylated RPL10, PANC-1 cell lysates were used for Co-IP with anti-UFSP2 antibody. As shown in Fig. 2C and Supplemental Fig. S3I, RPL10 was pulled down by UFSP2, indicating that UFSP2 directly binds with ufmylated RPL10. Since UFSP2 was unable to bind unmodified RPL10, Co-IP of RPL10 with anti-UFSP2 antibody to produce two modified bands was expected. Meanwhile, when UFSP2 was overexpressed, the level of RPL10 ufmylation was notably reduced (Fig. 2D and Supplemental Fig. S3J).

### RPL10 ufmylation affected the proliferation and colony formation of pancreatic cancer cells

To determine the effects of RPL10 ufmylation on pancreatic cancer cells, cell proliferation and colony-forming assay were performed after UFL1 knockdown in PANC-1 and Mia PaCa-2 cells. As shown in Fig. 2E, knockdown of UFL1 attenuated the proliferation rates of PANC-1 and Mia PaCa-2 cells. Moreover, the decrease of colony formation was obviously observed after the knockdown of UFL1 in PANC-1 cells (Fig. 2G). Similar situation was seen in Mia PaCa-2 cells as well (Fig. 2G).

Conversely, to test the participation of USFP2 in the modulation of cell proliferation and colony formation of the pancreatic cancer cells, UFSP2 was overexpressed in PANC-1 and Mia PaCa-2 cells, and cell growth-related characteristics were analyzed. After the overexpression of UFSP2, the rates of cell proliferation were decreased (Fig. 2F). Meanwhile, compared to pcDNA3.1 empty vector, overexpression of UFSP2 markedly reduced colony formation of PANC-1 and Mia PaCa-2 cells (Fig. 2H).

Given the close relationship between colony formation and cell stemness [36], these results pointed to a possible connection of RPL10 ufmylation with the changes of cell stemness.

### RPL10 ufmylation enhanced the stemness of pancreatic cancer cells

**Fig. 3:**
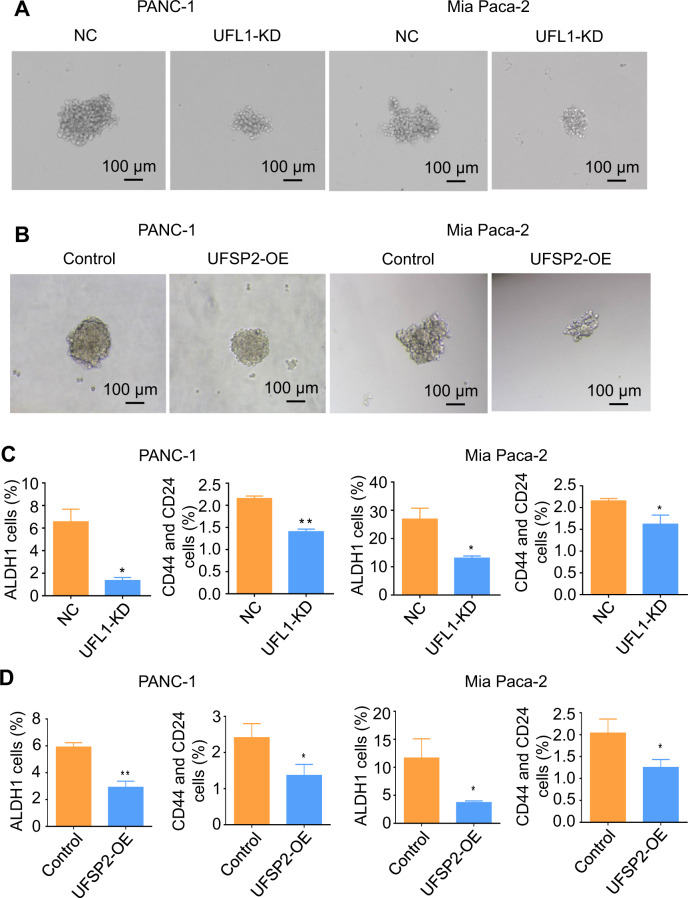
The relationship between ufmylation of RPL10 and the stemness of PANC-1 cells. **A** Sphere formation of PANC-1 cells and Mia PaCa-2 cells after the knockdown of UFL1. **B** Sphere formation of PANC-1 cells and Mia PaCa-2 cells after the overexpression of UFSP2. **C** The populations of ALDH1^+^ cells and CD44^+^-CD24^+^ cells in PANC-1 and Mia PaCa-2 cells after the knockdown of UFL1. The populations were analyzed by flow cytometry (*n* = 3). **D** The populations of ALDH1^+^ cells and CD44^+^-CD24^+^ cells in PANC-1 and Mia PaCa-2 cells after overexpressing UFSP2. The populations were analyzed by flow cytometry (*n* = 3). All experiments were independently repeated for 3 times, and the data are expressed as mean ± SD. ^*^P < 0.05 indicates statistically significant versus negative control. ^**^P < 0.01 indicates statistically very significant versus negative control.

To examine whether RPL10 ufmylation is related to cell stemness, sphere-forming assay, a typical method for measuring cell stemness, was conducted to observe the influences from the knockdown of UFL1 and the overexpression of UFSP2 in PANC-1 and Mia PaCa-2 cells. Consequently, the knockdown of UFL1 dramatically decreased the sizes of spheres for these cells (Fig. 3A). In accordance, the sizes of spheres were also decreased when UFSP2 was overexpressed in PANC-1 and Mia PaCa-2 cells (Fig. 3B).

Because aldehyde dehydrogenase 1 (ALDH1), CD44 and CD24 are well recognized cell surface markers for the stemness of pancreatic cancer cells, we next used these molecules as indicators to examine cell stemness. When antibodies against ALDH1-PE, CD44-APC and CD24-FITC were respectively used for the analysis by flow cytometry, the populations of ALDH^+^ and CD44^+^-CD24^+^ cells from UFL1 knockdown were markedly less compared to negative control (Fig. 3C). Similarly, the populations of ALDH^+^ and CD44^+^-CD24^+^ cells with UFSP2 overexpression were substantially lower than the cells with empty vector (Fig. 3D). Together, these results strongly indicated that PRL10 ufmylation resulted in the enhancement of cell stemness.

### RPL10 ufmylation promoted KLF4 expression for the stemness of pancreatic cancer cells

**Fig. 4:**
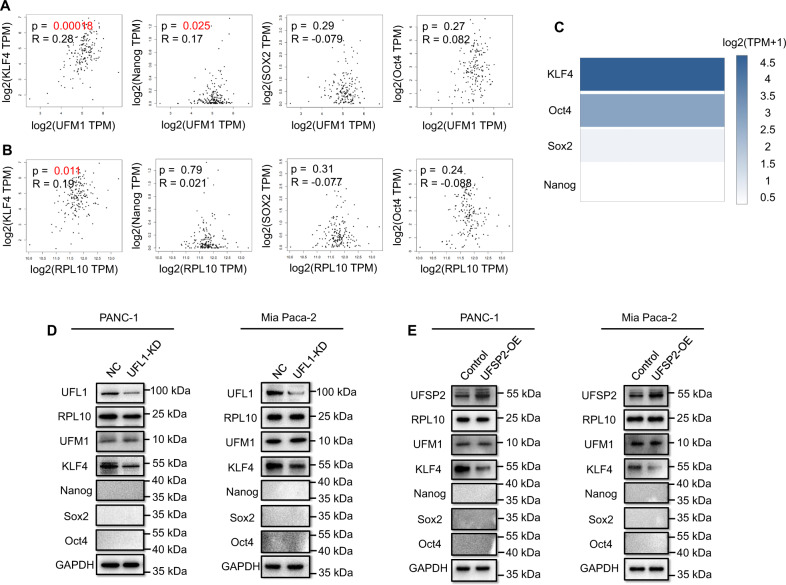
Identification of transcription factor KLF4 in PRL10 ufmylation related stemness of pancreatic cancer cells. **A** The correlation between stemness related transcription factors and UFM1 in tissues of PAAD patients (http://gepia2.cancer-pku.cn/#correlation). **B** The correlation between stemness related transcription factors and RPL10 in tissues of PAAD patients (http://gepia2.cancer-pku.cn/#correlation). **C** The relationship between the development of PAAD and gene transcripts of transcription factors (http://gepia2.cancer-pku.cn/#analysis). TPM is the abbreviation of transcripts per million of genes. **D** Protein expression of different transcription factors after the knockdown of UFL1 in PANC-1 and Mia PaCa-2 cells. **E** Protein expression of different transcription factors after the overexpression of UFSP2 in PANC-1 and Mia PaCa-2 cells. All experiments were independently repeated for 3 times.

To identify potential molecules to regulate the stemness from RPL10 ufmylation, we chose four well-known stemness related transcription factors, including KLF4, Nanog, Oct4 and SOX-2, to analyze the correlation between these transcription factors and RPL10 or UFM1 in the tissues of PAAD patients (http://gepia2.cancer-pku.cn/#correlation). The expression of KLF4 was positively correlated with both UFM1 (Fig. 4A) and RPL10 (Fig. 4B), reflecting the potential correlation of KLF4 with RPL10 ufmylation. The expression of Nanog was correlated with UFM1 (Fig. 4A) but not RPL10 (Fig. 4B), whereas Sox2 and Oct4 showed no relationships with either RPL10 or UFM1 (Fig. 4A, B). We then analyzed their association with PAAD development by gene transcripts using multiple gene analysis in the database (http://gepia2.cancer-pku.cn/#analysis). As a result, KLF4 was a predominant factor to associate with PAAD development (Fig. 4C). Next, to verify the involvement of these transcription factors, their protein expressions, after the knockdown of UFL1 and the overexpression of UFSP2, were determined by immunoblotting. Among these transcription factors, only KLF4 was significantly decreased with the knockdown of UFL1 in both PANC-1 and Mia-Paca-2 cells (Fig. 4D and Supplemental Fig. S3K). Surprisingly, the protein expressions of Nanog, Oct4 and Sox2 were not detectable by Western blotting in both PANC-1 and Mia-Paca-2 cells, and even in the negative control (Fig. 4D and Supplemental Fig. S3K), due possibly to extremely low abundance of Nanog, Oct4 and Sox2 in the cells. When UFSP2 was overexpressed in PANC-1 and Mia-Paca-2 cells, the reduction of KLF4 was also observed (Fig. 4E and Supplemental Fig. S3L). Collectively, these results indicated a close relationship between KLF4 and RPL10 ufmylation.

### RNA interference of UFL1 exerted anti-tumor activity in xenografted mouse model

**Fig. 5:**
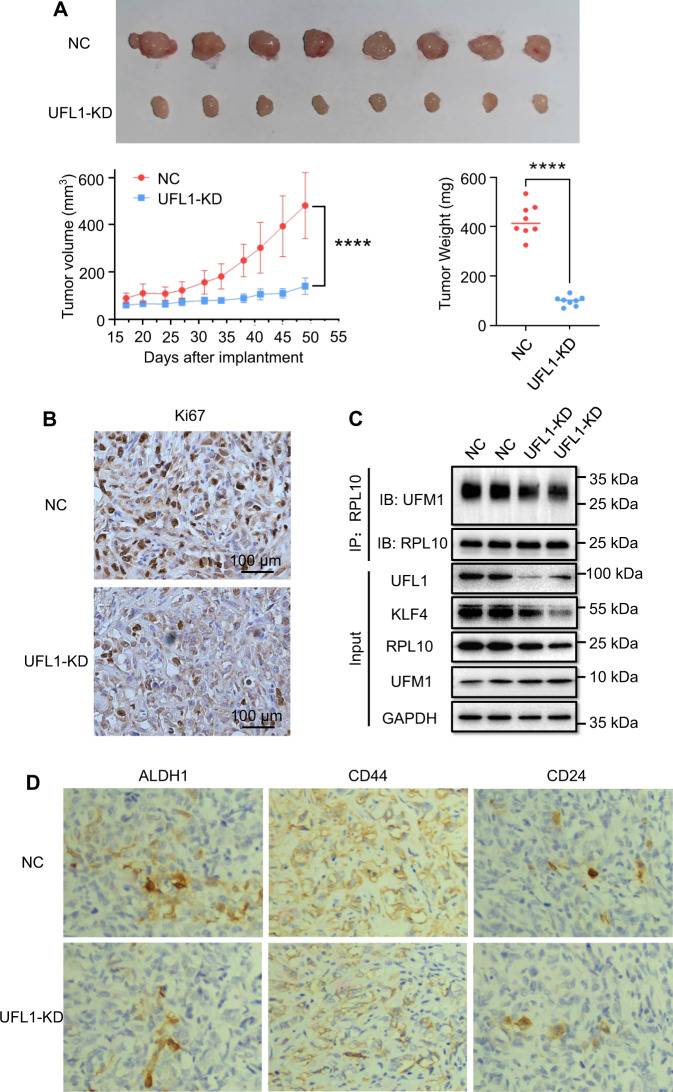
Confirmation of the involvement of UFL1 in tumorigenesis and cell stemness in vivo. **A** The effects of the knockdown of UFL1 on tumor growth. Each group contained 8 mice, and data are expressed as mean ± SD. ^*^P < 0.05 indicates statistically significant versus negative control. ^**^P < 0.01 indicates statistically very significant versus negative control. **B** The effect of UFL1 knockdown on tumor growth by Ki67 staining. **C** Examination of RPL10 ufmylation and KLF4 expression in tumor tissues by Co-IP and WB. Tumor tissues were randomly chosen from each group. **D** The effects of the knockdown of UFL1 on cell stemness related markers by immunochemical staining.

To ascertain the association of RPL10 ufmylation and PAAD development in vivo, stable PANC-1 cell lines with UFL1 knockdown were established, and the cells were then subcutaneously implanted in the flank region of NPG mice to examine tumor formation. As anticipated, knockdown of UFL1 significantly inhibited the growth of the xenografts (Fig. 5A). Coincidently, the Ki67 staining showed a decline of tumor growth with UFL1 knockdown compared to NC group (Fig. 5B). When tumor tissues were analyzed, the level of ufmylated RPL10 was markedly decreased from UFL1 knockdown, along with the decline of KLF4 (Fig. 5C and Supplemental Fig. S3M). Furthermore, based on immunohistochemical staining with respective antibody, UFL1 knockdown showed a significant decline of the populations of ALDH1^+^, CD44^+^ and CD24^+^ cells (Fig. 5D). These data from xenografted mouse model clearly demonstrated that RPL10 ufmylation is associated with tumorigenesis through the increase of cell stemness.

### Mutagenesis of ufmylation sites in RPL10 influenced the proliferation and stemness of pancreatic cancer cells

**Fig. 6:**
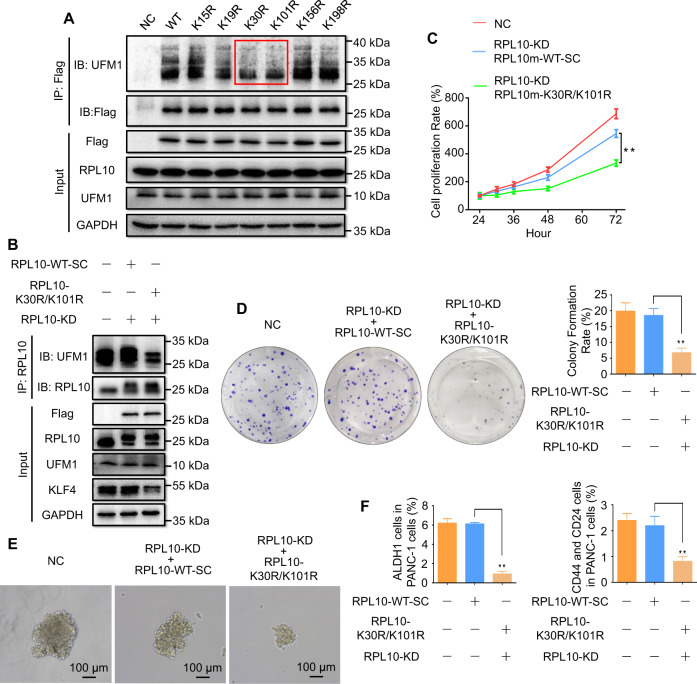
The effects of RPL10 ufmylation on proliferation and stemness of pancreatic cancer cells by the mutagenesis of ufmylation sites. **A** Identification of ufmylation sites in RPL10 with different mutants. **B** The change of RPL10 ufmylation and KLF4 level after the knockdown of endogenous RPL10 and the overexpression of RPL10 mutants in PANC-1 cells. **C** The change of cell proliferation after the knockdown of endogenous RPL10 and the overexpression of RPL10 mutants in PANC-1 cells. The proliferation rates were detected by CCK-8 method (*n* = 10). **D** The change of colony formation of PANC-1 cells after the knockdown of endogenous RPL10 and the overexpression of RPL10 mutants. The colonies were stained with crystal violet and analyzed (*n* = 3). **E** The change of sphere formation of PANC-1 cells after the knockdown of endogenous RPL10 and the overexpression of RPL10 mutants. **F** The populational change of ALDH1^+^ cells and CD44^+^-CD24^+^ in PANC-1 cells after the knockdown of endogenous RPL10 and the overexpression of RPL10 mutants. Cell populations were analyzed by flow cytometry (*n* = 3). All experiments were independently repeated for 3 times, and the data are expressed as mean ± SD. ^*^P < 0.05 indicates statistically significant versus negative control. ^**^P < 0.01 indicates statistically very significant versus negative control.

To identify the modification sites that conjugate UFM1 onto RPL10, the precipitates of RPL10 and UFM1 from Co-IP (NC, Fig. 2B and Supplemental Fig. S3H) was hydrolyzed by trypsin and then analyzed by mass spectrometry (MS). Given that the specific modification of Lys residue conjugating the unique dipeptide (Val-Gly) produces a signature tripeptide Lys-Gly-Val (KGV) with a mass increase of 156.09 Da, MS/MS analyses of the peptide fragments from trypsin digestion suggested that RPL10 is potentially modified at 6 different sites (Supplemental Fig. S2A and Supplemental Fig. S2B). Next, to rule out false positives from MS/MS analyses and to verify modification sites in RPL10, each Lys residue was mutated to Arg to generate mutants of K15R, K19R, K30R, K101R, K156R and K198R by site-directed mutagenesis. The mutants and wild type (WT) of RPL10 with 3x flag tag on their C-termini were introduced into lentiviral expression vectors and then transfected into HEK293T cells for the generation of lentiviral particles. The recombinant lentiviruses were transfected into PANC-1 cells for the overexpression of RPL10 and its mutants. After evaluating the ufmylation levels of RPL10 and its mutants by Co-IP and Western blot, the mutants of K30R and K101R noticeably showed lower modification than WT and other mutants (Fig. 6A and Supplemental Fig. S3N), confirming that K30 and K101 are the actual sites for ufmylation in RPL10.

To further examine the effects of RPL10 ufmylation on cell proliferation, colony formation and stemness, the mutants of K30R/K101R or WT with synonymous codons (WT-SC) for RNAi target (Supplemental Fig. S2C) were packed as lentiviral particles for overexpression. After knockdown of endogenous RPL10 to eliminate possible interference from the change of RPL10 expression, RPL10-WT-SC and RPL10-K30R/K101R mutants were overexpressed in PANC-1 cells, respectively. As shown in Fig. 6B and Supplemental Fig. S3O, K30R/K101R mutants significantly decreased RPL10 ufmylation, along with the decline of KLF4. Moreover, cell proliferation (Fig. 6C) and colony formation (Fig. 6D) were significantly reduced by the mutants of K30R and K101R. Additionally, both mutants decreased the sizes of the spheres (Fig. 6E), and the populations of ALDH^+^ and CD44^+^-CD24^+^ cells were also markedly less compared to RPL10-WT-SC (Fig. 6F).

## Discussion

PAAD, the most malignant cancer with high mortality, is usually diagnosed at late stage of the disease and not suitable for surgery [1, 34]. Although PAAD can be moderately ameliorated with a variety of treatment strategies in recent years, the survival time of PAAD patients remains shorter than any other types of cancer [35]. In addition, high resistance rates against chemotherapeutic agents, such as gemcitabine, further increases the difficulty on the treatment of PAAD patients [4]. Although tremendous efforts have been made to prolong the survival time of PAAD patients, the situation has not become significantly better due mainly to the lack of novel molecular targets and therapeutic strategies.

Apart from ribosome biogenesis and protein synthesis, RPL10 is involved in several cellular processes, such as metabolic regulation, differentiation, proliferation and apoptosis [20]. Previously, in addition to the close relationship between RPL10 R98S mutation and leukemia [26–28], the expression of PRL10 has been indicated to affect the development of various cancers, including prostate cancer, ovarian cancer and pancreatic cancer [21–25, 29]. In our previous study, RPL10 was also found to regulate IKKγ and p65 for inhibiting the proliferation of pancreatic cancer cells [29]. Despite the association between RPL10 expression and cancer development in the literatures [20], whether post-translational modifications of RPL10 plays a role in cancer development remains poorly understood. Given that RPL26 is identified as an ufmylation target by UFM1-captured proteomics analysis [15, 16], it would be interesting to see if RPL10 ufmylation really occurs in pancreatic cancer cells, what enzyme system would be responsible for this modification, whether RPL10 ufmylation is associated with cell stemness and whether interference of RPL10 ufmylation would exhibit antitumor activity. To address these important questions, the present study was naturally focused on the confirmation of RPL10 ufmylation and its relationship with PAAD development.

To ensure the association between RPL10 ufmylation and PAAD with clinical relevance, we first determined the specificity of RPL10 ufmylation in pancreatic cancer cell lines. RPL10 ufmylation was clearly observed in pancreatic cancer cells, whereas another ribosomal protein, RPS3, was unable to be modified by UFM1. Then, the difference on RPL10 ufmylation between tumor tissues and adjacent normal tissues from PAAD patients was examined, and higher level of RPL10 ufmylation was found in tumor tissues of PAAD patients.

To elucidate the process of RPL10 ufmylation involved in pancreatic cancer cells, we used immunoprecipitation to examine the interactions between co-substrates RPL10 and UFM1 as well as between RPL10 and E3 ligase UFL1, which could be similar to the ufmylating modifications of ASC1 and RPL26 [12, 15, 16]. Consequently, RPL10 was indeed ufmylated with the conjugation of UFM1. Therefore, the ufmylating modification of RPL10 was unambitiously confirmed by both the attachment of UFM1 and the cleavage by UFSP2, indicating that the ufmylating modification of RPL10 is mediated by a cascade system [12].

Because RPL10 ufmylation was associated with PAAD development and originally reported in E14 embryotic stem cells [12], it was reasonable for us to hypothesize that RPL10 ufmylation might be related to the proliferation and stemness of pancreatic cancer cells for tumor development. Therefore, we knocked down E3 ligase UFL1 to evaluate the changes of RPL10 ufmylation, proliferation and stemness-related characteristic features in pancreatic cancer cells. Consequently, the reduction of RPL10 ufmylation truly decreased the proliferation and stemness of the cells. To the best of our knowledge, this is the first time to provide solid evidence on the modulation of cell stemness by ufmylation modification. Meanwhile, when UFSP2, the protease responsible for the cleavage of UFM1, was overexpressed, exactly same outcomes were observed in pancreatic cancer cells, which provides an additional line of evidence.

Next, to find out the underlying player(s) related to the change of cell stemness, we chose four major transcription factors that are commonly related to the stemness of pancreatic cancer cells [37–39], including KLF4, SOX2, Nanog and Oct4, to identify potential molecules for the regulation of the stemness. Among them, transcription level of KLF4 was positively correlated with both co-substrates of RPL10 and UFM1. Additionally, the protein expression of KLF4 showed a remarkable decline after UFL1 knockdown or UFSP2 overexpression. Since KLF4 plays an important role in maintaining the stemness and self-renewal propensity of pancreatic cancer cells and promoting the progression of PAAD [40], it could be a key transcription factor in the regulation of ufmylation for the stemness of pancreatic cancer cells. In contrast, the protein expressions of SOX2, Nanog and Oct4 were under detection limit in our experiments. To examine the influence of RPL10 ufmylation on tumorigenesis, pancreatic cancer cells with stable knockdown of UFL1 were implanted in immune-deficient mice to evaluate tumor growth. Tumor sizes of UFL1 knockdown were significantly smaller than control group with the decrease of RPL10 ufmylation in tumor tissues. Meanwhile, the transcription factor KLF4 was much lower after the knockdown of UFL1, and the populations of ALDH1^+^, CD44^+^ and CD24^+^ cells decreased accordingly by immunochemical staining. These results have strongly indicated that RPL10 ufmylation can enhance cell stemness for PAAD development.

Because the regulation of ufmylation by both UFL1 and UFSP2 could be interfered from various substrates that are also ufmylated, the mutations of potential ufmylation sites in the substrate are necessary for the elucidation of consequent cellular functions [41]. To identify the sites for ufmylating modification in RPL10, we first obtained potential ufmylation sites of RPL10 by MS/MS analyses. Among these sites, the mutations of Lys30 and Lys101 obviously decreased RPL10 ufmylation. To ascertain the modification sites for biochemical dissection of the functions of RPL10 ufmylation in pancreatic cancer cells, we overexpressed RPL10-K30R/K101R mutants to examine the changes of RPL10 ufmylation after the elimination of potential interferences from endogenous RPL10. The mutations of K30R/K101R indeed decreased the level of RPL10 ufmylation and significantly inhibited the proliferation and the stemness of PANC-1 cells, further confirming that actual ufmylation modifications took place at the sites of K30 and K101 in RPL10 for functional impacts.

In summary, we have confirmed the ufmylation of RPL10 in pancreatic cancer cells, xenografted tumor tissues as well as tumor tissues from PAAD patients. RPL10 ufmylation has been demonstrated to associate with cell stemness and PAAD development. Therefore, the interference of RPL10 ufmylation might represent a new therapeutic strategy of PAAD. This study has not only generated new insights into the extra-ribosomal functions of RPL10, but also shed a light on potential treatment of PAAD.

## Supplementary information

Supplementary Material-Table

Supplementary Material-Figures and original western blots

Checklist

## Data Availability

Please contact the author for data requests.
