# Supplementary material for: The ufmylation modification of ribosomal protein L10 in the development of pancreatic adenocarcinoma
Source: Cell Death Dis. 2023 Jun 7;14(6):350. doi: 10.1038/s41419-023-05877-y (PMC10244432; doi:10.1038/s41419-023-05877-y)
Supplement: Supplementary file 1 — Supplementary Material-Table [file 41419_2023_5877_MOESM1_ESM.docx]

**Supplementary Materials**

**Supplementary Table**

**Table S1 Nucleotide sequences for RNA interference**

| Gene | Sequence for RNAi |
| --- | --- |
| UFL1 | GAGCGAAGAAGGAAAGCAA |
| RPL10 | GCCAAUAAGUACAUGGUAA |
