# Supplementary material for: The ufmylation modification of ribosomal protein L10 in the development of pancreatic adenocarcinoma
Source: Cell Death Dis. 2023 Jun 7;14(6):350. doi: 10.1038/s41419-023-05877-y (PMC10244432; doi:10.1038/s41419-023-05877-y)
Supplement: Supplementary file 2 — Supplementary Material-Figures and original western blots [file 41419_2023_5877_MOESM2_ESM.pptx]

## Slide 1
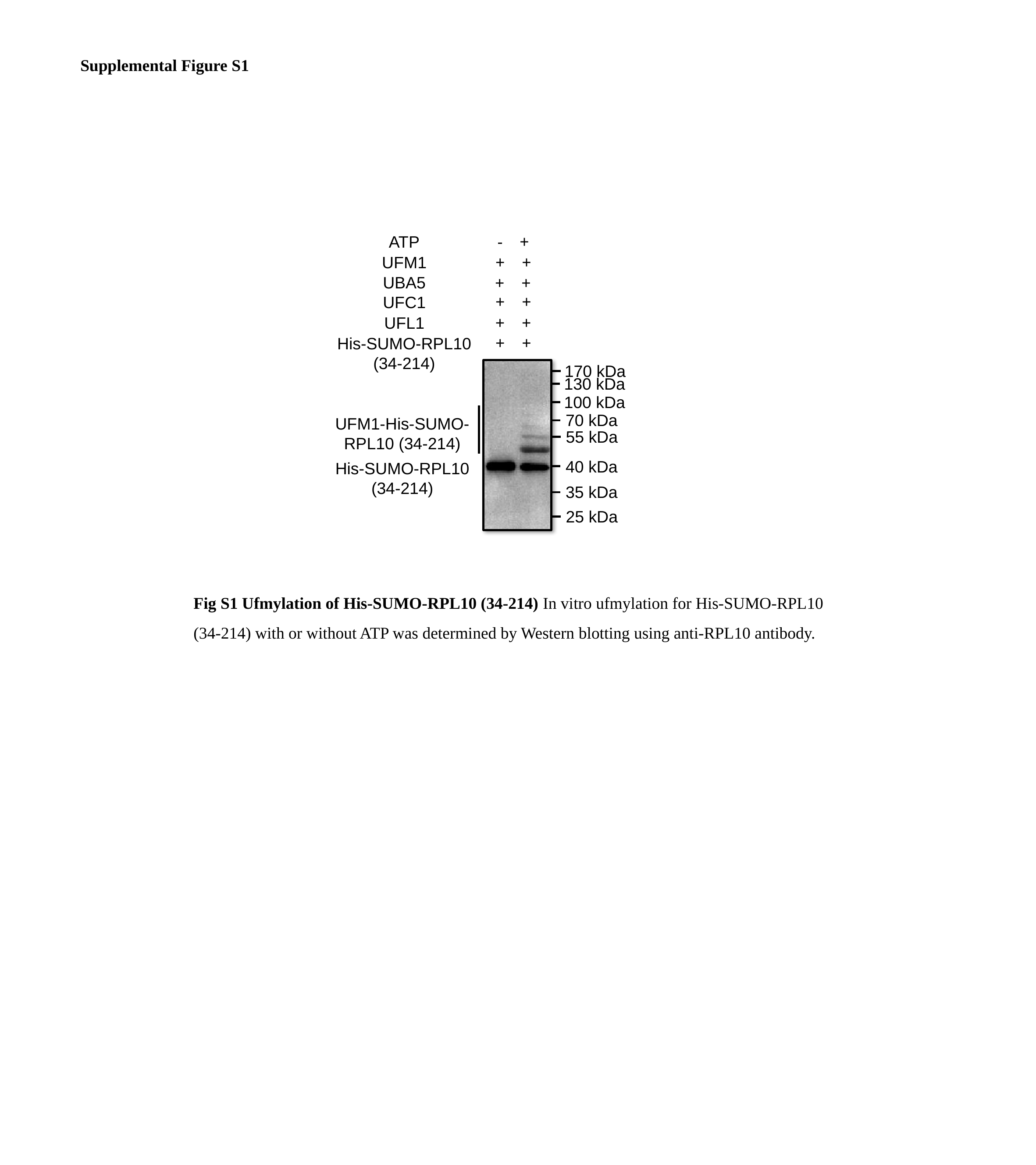

Supplemental Figure S1
- +
ATP
UFM1
+ +
UBA5
+ +
+ +
UFC1
UFL1
+ +
+ +
His-SUMO-RPL10 (34-214)
170 kDa
130 kDa
100 kDa
70 kDa
UFM1-His-SUMO-RPL10 (34-214)
55 kDa
40 kDa
His-SUMO-RPL10 (34-214)
35 kDa
25 kDa
Fig S1 Ufmylation of His-SUMO-RPL10 (34-214) In vitro ufmylation for His-SUMO-RPL10 (34-214) with or without ATP was determined by Western blotting using anti-RPL10 antibody.

## Slide 2
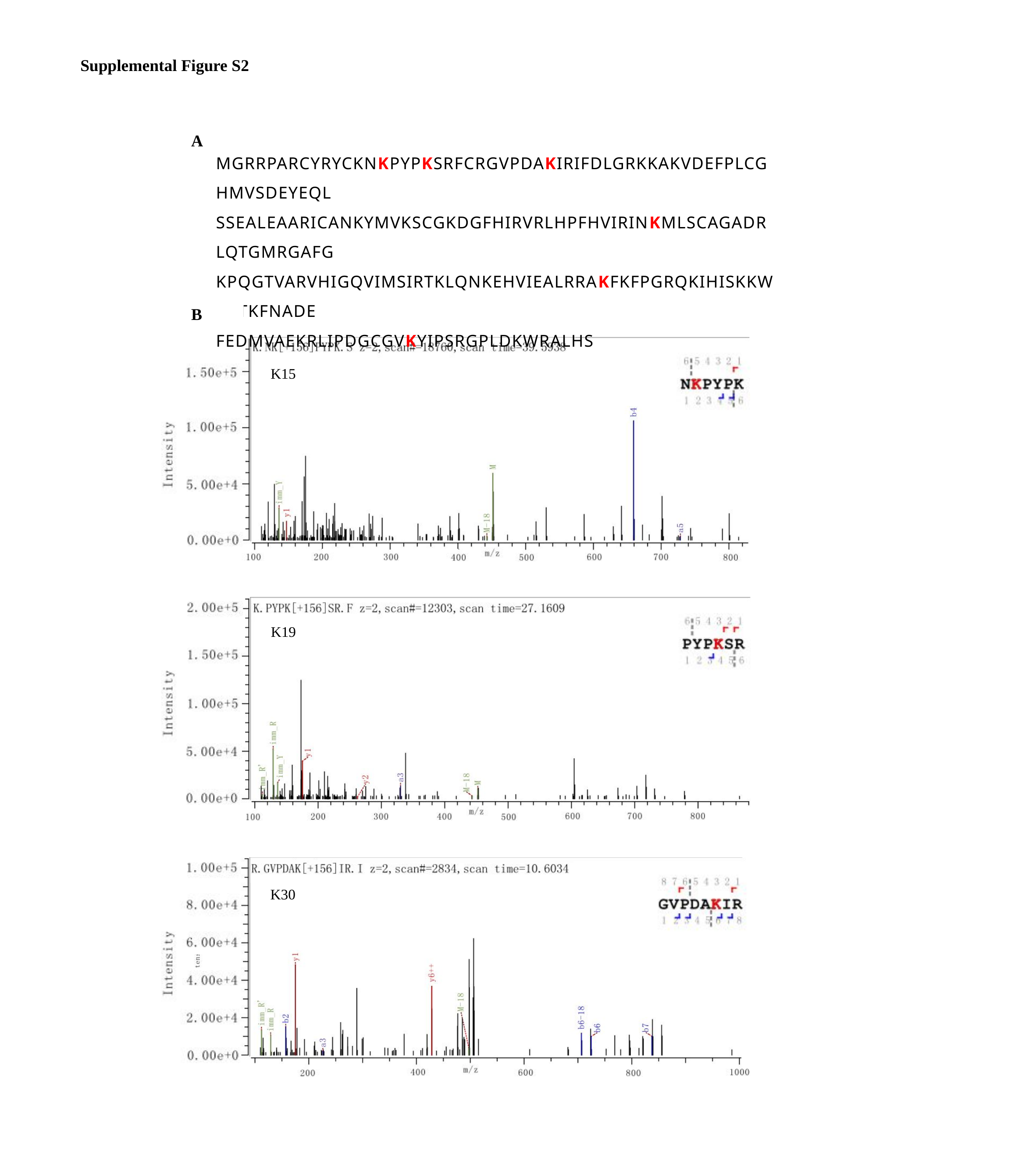

Supplemental Figure S2
A
MGRRPARCYRYCKNKPYPKSRFCRGVPDAKIRIFDLGRKKAKVDEFPLCGHMVSDEYEQL
SSEALEAARICANKYMVKSCGKDGFHIRVRLHPFHVIRINKMLSCAGADRLQTGMRGAFG
KPQGTVARVHIGQVIMSIRTKLQNKEHVIEALRRAKFKFPGRQKIHISKKWGFTKFNADE
FEDMVAEKRLIPDGCGVKYIPSRGPLDKWRALHS
B
K15
K19
K30

## Slide 3
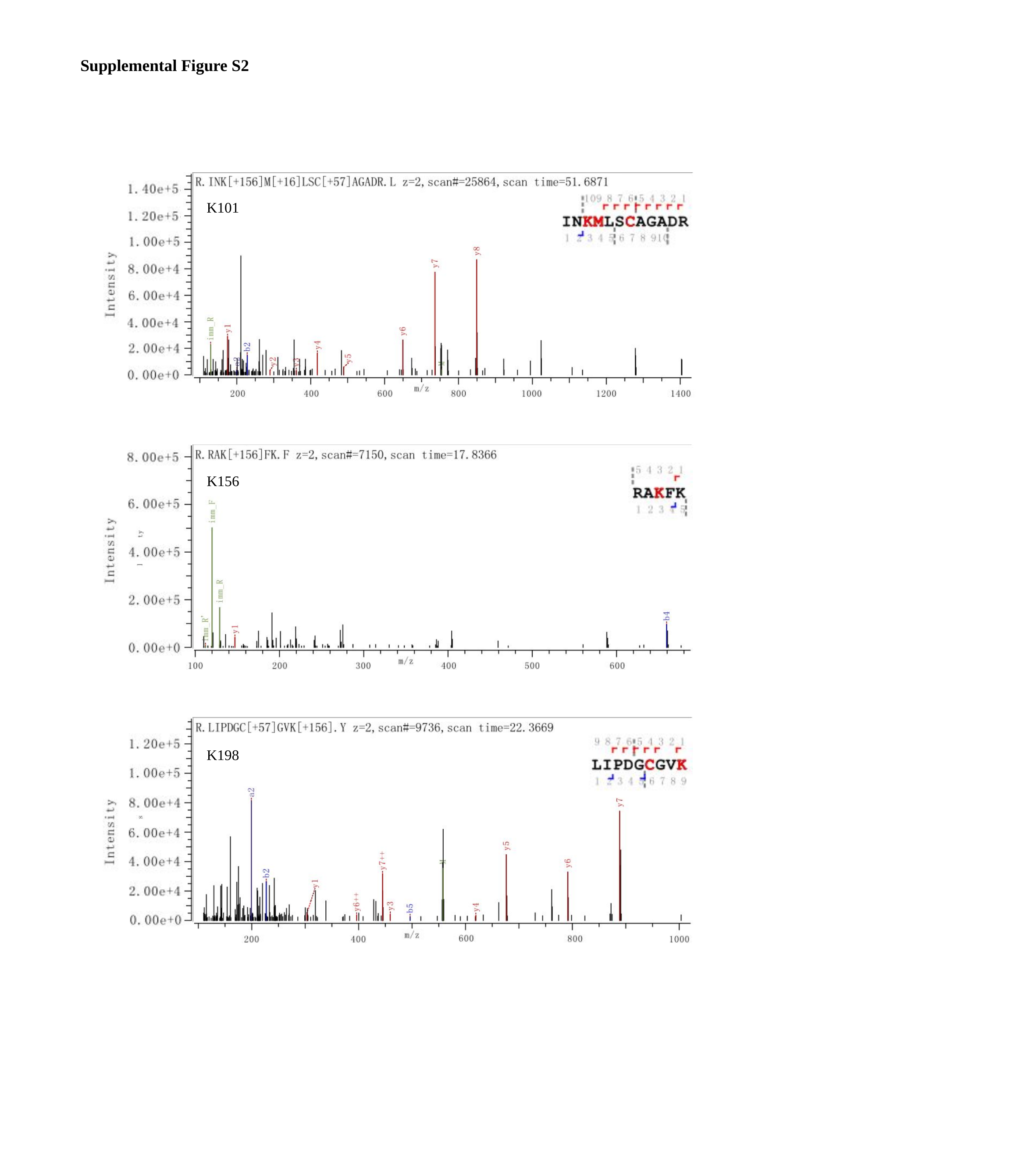

Supplemental Figure S2
K101
K156
K198

## Slide 4
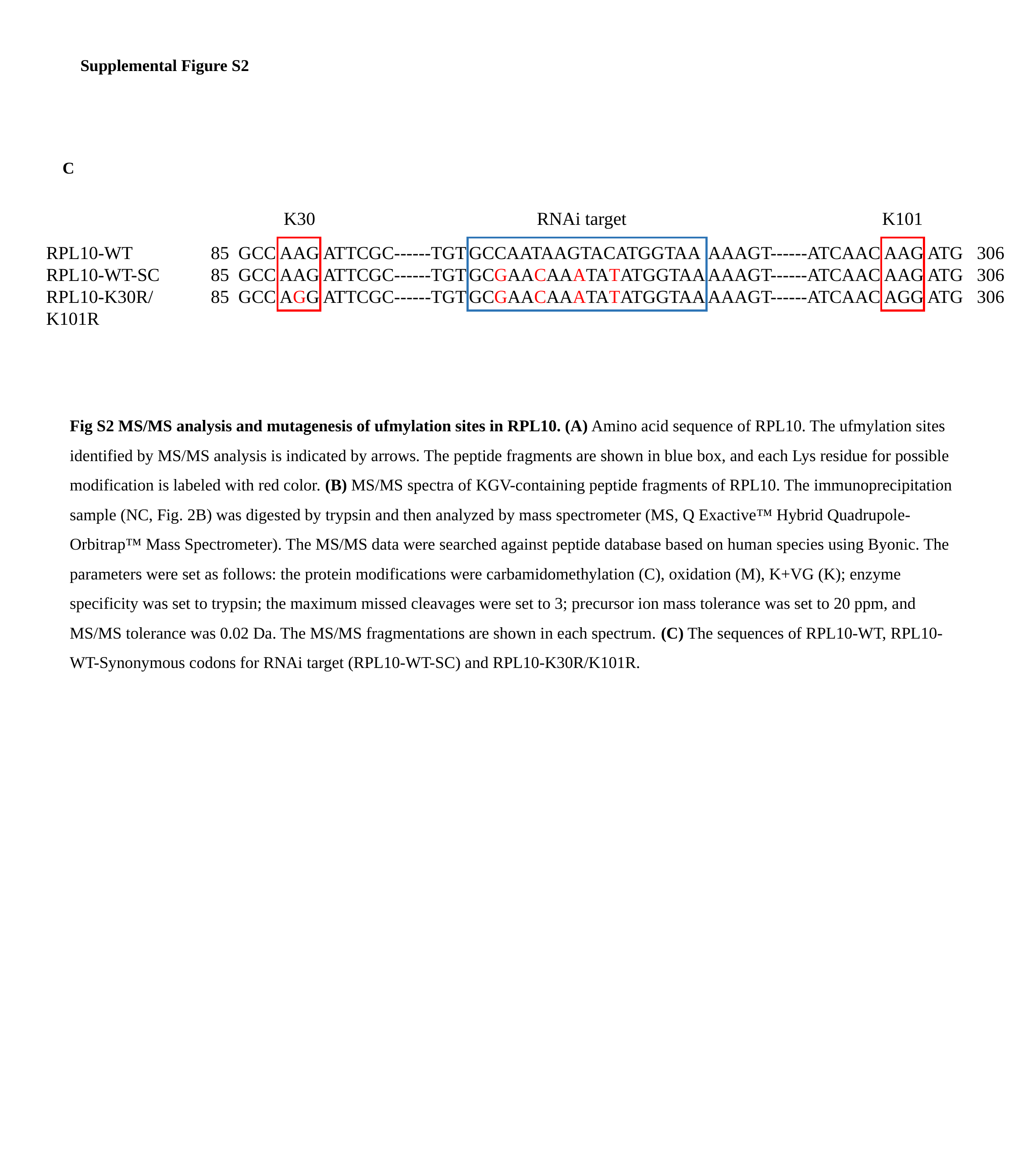

Supplemental Figure S2
C
K30
RNAi target
K101
RPL10-WT
RPL10-WT-SC
RPL10-K30R/K101R
85 GCC AAG ATTCGC------TGT
85 GCC AAG ATTCGC------TGT
85 GCC AGG ATTCGC------TGT
GCCAATAAGTACATGGTAA AAAGT------ATCAAC AAG ATG 306
GCGAACAAATATATGGTAA AAAGT------ATCAAC AAG ATG 306 GCGAACAAATATATGGTAA AAAGT------ATCAAC AGG ATG 306
Fig S2 MS/MS analysis and mutagenesis of ufmylation sites in RPL10. (A) Amino acid sequence of RPL10. The ufmylation sites identified by MS/MS analysis is indicated by arrows. The peptide fragments are shown in blue box, and each Lys residue for possible modification is labeled with red color. (B) MS/MS spectra of KGV-containing peptide fragments of RPL10. The immunoprecipitation sample (NC, Fig. 2B) was digested by trypsin and then analyzed by mass spectrometer (MS, Q Exactive™ Hybrid Quadrupole-Orbitrap™ Mass Spectrometer). The MS/MS data were searched against peptide database based on human species using Byonic. The parameters were set as follows: the protein modifications were carbamidomethylation (C), oxidation (M), K+VG (K); enzyme specificity was set to trypsin; the maximum missed cleavages were set to 3; precursor ion mass tolerance was set to 20 ppm, and MS/MS tolerance was 0.02 Da. The MS/MS fragmentations are shown in each spectrum. (C) The sequences of RPL10-WT, RPL10-WT-Synonymous codons for RNAi target (RPL10-WT-SC) and RPL10-K30R/K101R.

## Slide 5
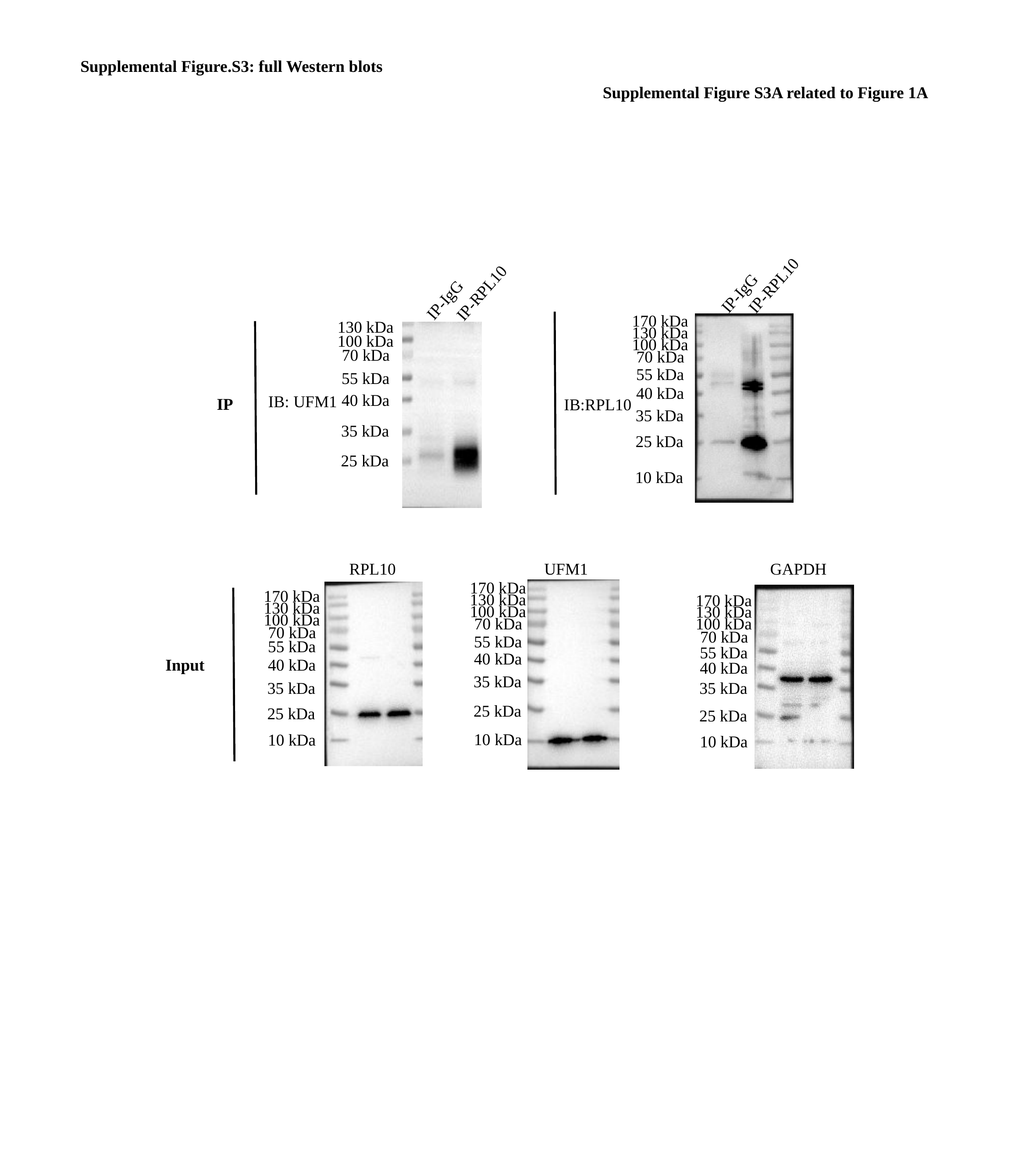

Supplemental Figure.S3: full Western blots
Supplemental Figure S3A related to Figure 1A
IP-RPL10
IP-RPL10
IP-IgG
IP-IgG
170 kDa
130 kDa
130 kDa
100 kDa
100 kDa
70 kDa
70 kDa
55 kDa
55 kDa
40 kDa
40 kDa
IB: UFM1
IP
IB:RPL10
35 kDa
35 kDa
25 kDa
25 kDa
10 kDa
UFM1
GAPDH
RPL10
170 kDa
170 kDa
130 kDa
170 kDa
130 kDa
100 kDa
130 kDa
100 kDa
70 kDa
100 kDa
70 kDa
70 kDa
55 kDa
55 kDa
55 kDa
40 kDa
40 kDa
Input
40 kDa
35 kDa
35 kDa
35 kDa
25 kDa
25 kDa
25 kDa
10 kDa
10 kDa
10 kDa

## Slide 6
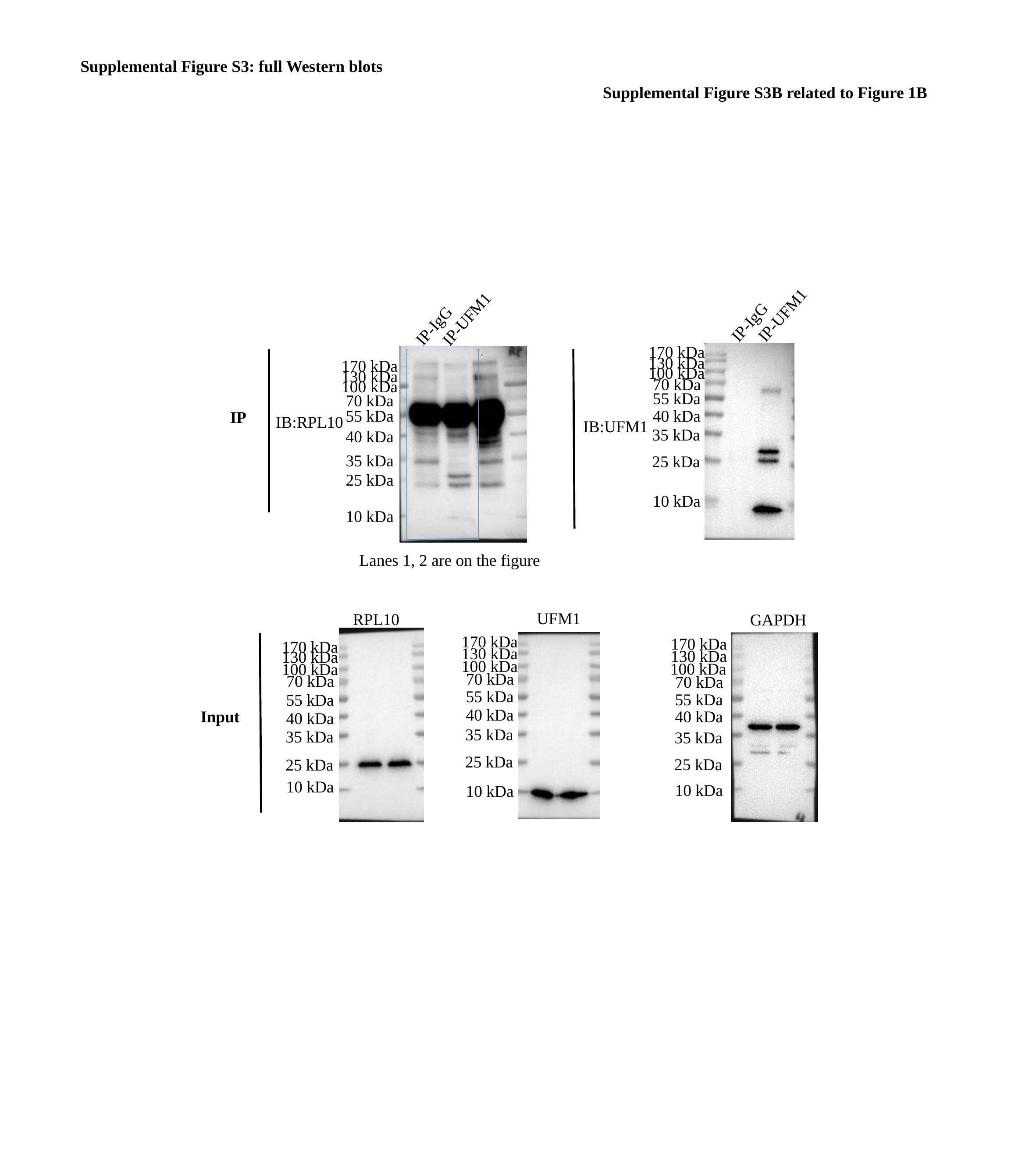

Supplemental Figure S3: full Western blots
Supplemental Figure S3B related to Figure 1B
IP-UFM1
IP-UFM1
IP-IgG
IP-IgG
170 kDa
130 kDa
100 kDa
70 kDa
55 kDa
40 kDa
IB:UFM1
35 kDa
25 kDa
10 kDa
170 kDa
130 kDa
100 kDa
70 kDa
55 kDa
IP
IB:RPL10
40 kDa
35 kDa
25 kDa
10 kDa
Lanes 1, 2 are on the figure
UFM1
RPL10
GAPDH
170 kDa
130 kDa
100 kDa
70 kDa
55 kDa
40 kDa
35 kDa
25 kDa
10 kDa
170 kDa
170 kDa
130 kDa
130 kDa
100 kDa
100 kDa
70 kDa
70 kDa
55 kDa
55 kDa
40 kDa
Input
40 kDa
35 kDa
35 kDa
25 kDa
25 kDa
10 kDa
10 kDa

## Slide 7
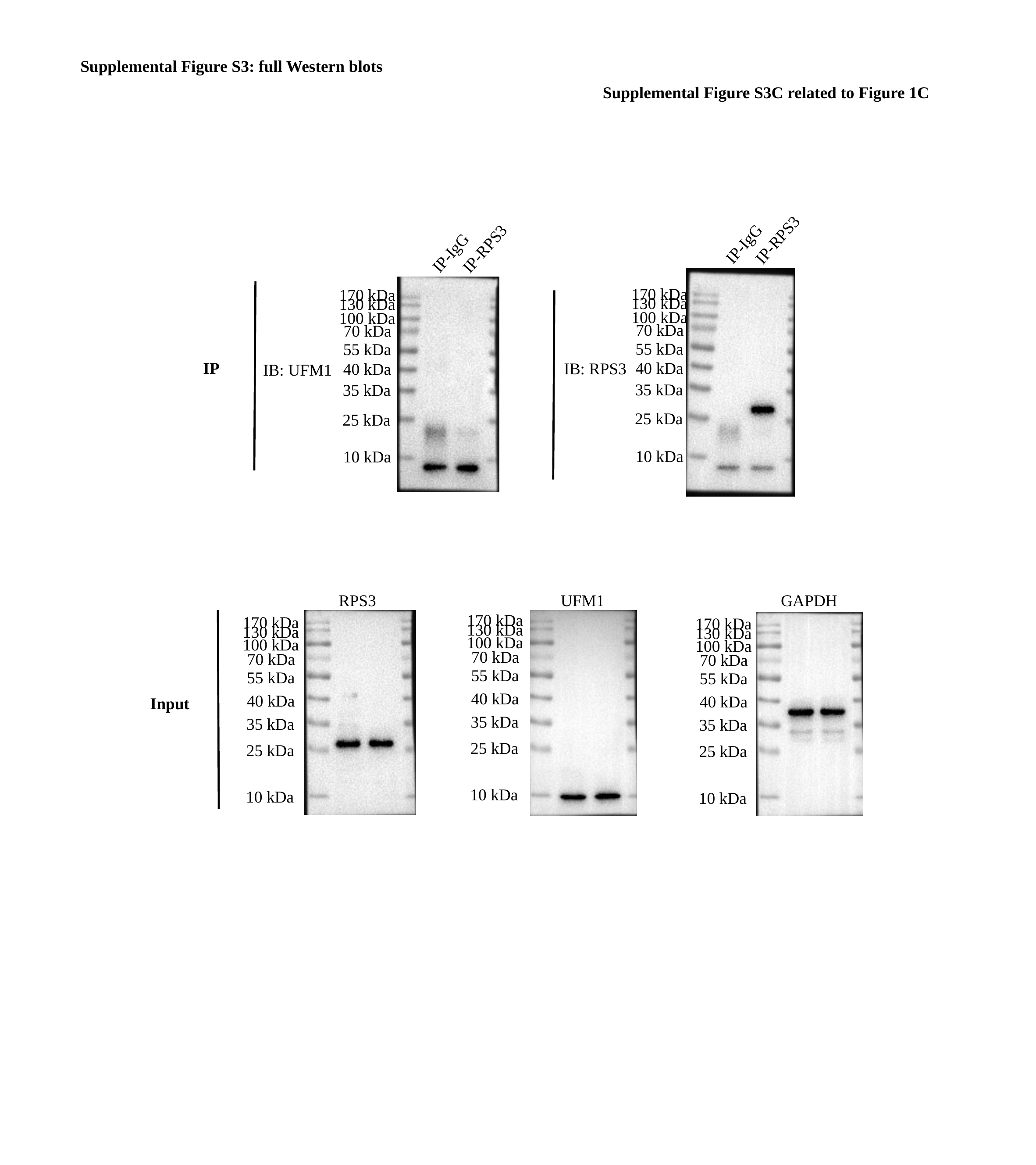

Supplemental Figure S3: full Western blots
Supplemental Figure S3C related to Figure 1C
IP-RPS3
IP-IgG
IP-RPS3
IP-IgG
170 kDa
170 kDa
130 kDa
130 kDa
100 kDa
100 kDa
70 kDa
70 kDa
55 kDa
55 kDa
IP
40 kDa
IB: RPS3
40 kDa
IB: UFM1
35 kDa
35 kDa
25 kDa
25 kDa
10 kDa
10 kDa
UFM1
GAPDH
RPS3
170 kDa
170 kDa
170 kDa
130 kDa
130 kDa
130 kDa
100 kDa
100 kDa
100 kDa
70 kDa
70 kDa
70 kDa
55 kDa
55 kDa
55 kDa
40 kDa
40 kDa
40 kDa
Input
35 kDa
35 kDa
35 kDa
25 kDa
25 kDa
25 kDa
10 kDa
10 kDa
10 kDa

## Slide 8
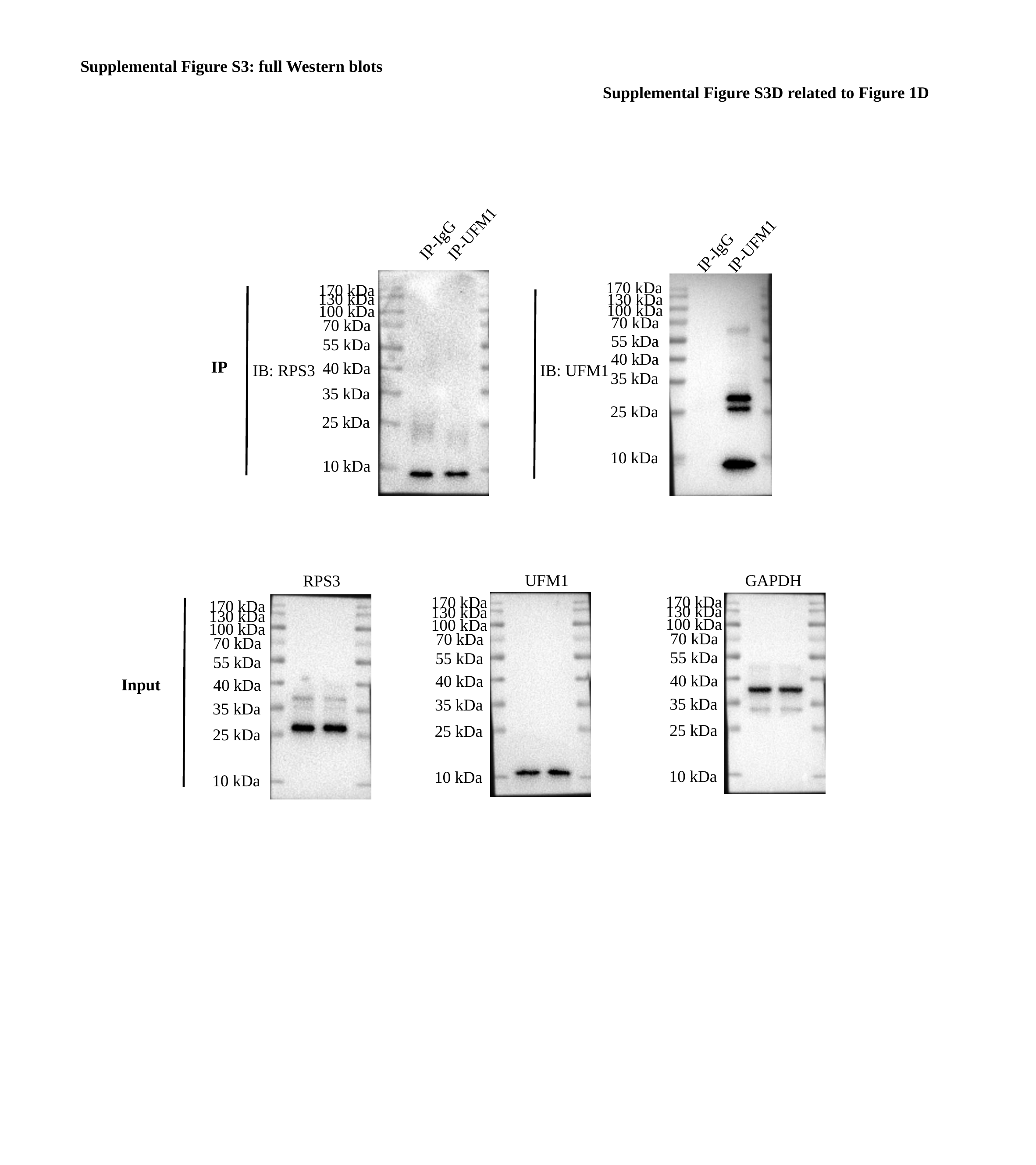

Supplemental Figure S3: full Western blots
Supplemental Figure S3D related to Figure 1D
IP-UFM1
IP-IgG
IP-UFM1
IP-IgG
170 kDa
170 kDa
130 kDa
130 kDa
100 kDa
100 kDa
70 kDa
70 kDa
55 kDa
55 kDa
40 kDa
IP
40 kDa
IB: UFM1
IB: RPS3
35 kDa
35 kDa
25 kDa
25 kDa
10 kDa
10 kDa
UFM1
GAPDH
RPS3
170 kDa
170 kDa
170 kDa
130 kDa
130 kDa
130 kDa
100 kDa
100 kDa
100 kDa
70 kDa
70 kDa
70 kDa
55 kDa
55 kDa
55 kDa
40 kDa
40 kDa
Input
40 kDa
35 kDa
35 kDa
35 kDa
25 kDa
25 kDa
25 kDa
10 kDa
10 kDa
10 kDa

## Slide 9
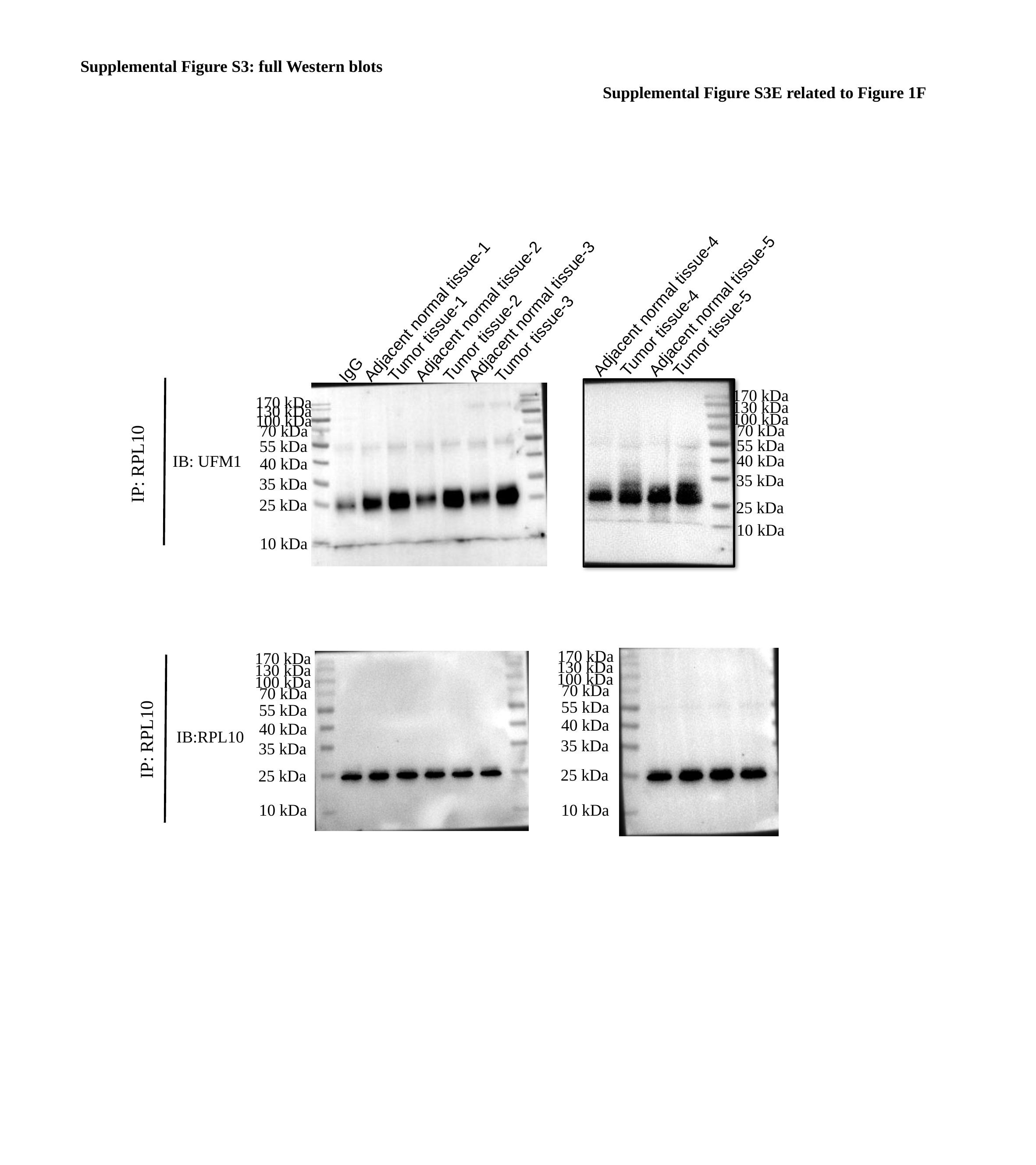

Supplemental Figure S3: full Western blots
Supplemental Figure S3E related to Figure 1F
Adjacent normal tissue-4
Adjacent normal tissue-5
Tumor tissue-4
Adjacent normal tissue-2
Tumor tissue-5
Adjacent normal tissue-1
Adjacent normal tissue-3
IgG
Tumor tissue-2
Tumor tissue-1
Tumor tissue-3
170 kDa
170 kDa
130 kDa
130 kDa
100 kDa
100 kDa
70 kDa
70 kDa
55 kDa
55 kDa
40 kDa
IB: UFM1
IP: RPL10
40 kDa
35 kDa
35 kDa
25 kDa
25 kDa
10 kDa
10 kDa
170 kDa
170 kDa
130 kDa
130 kDa
100 kDa
100 kDa
70 kDa
70 kDa
55 kDa
55 kDa
40 kDa
40 kDa
IB:RPL10
IP: RPL10
35 kDa
35 kDa
25 kDa
25 kDa
10 kDa
10 kDa

## Slide 10
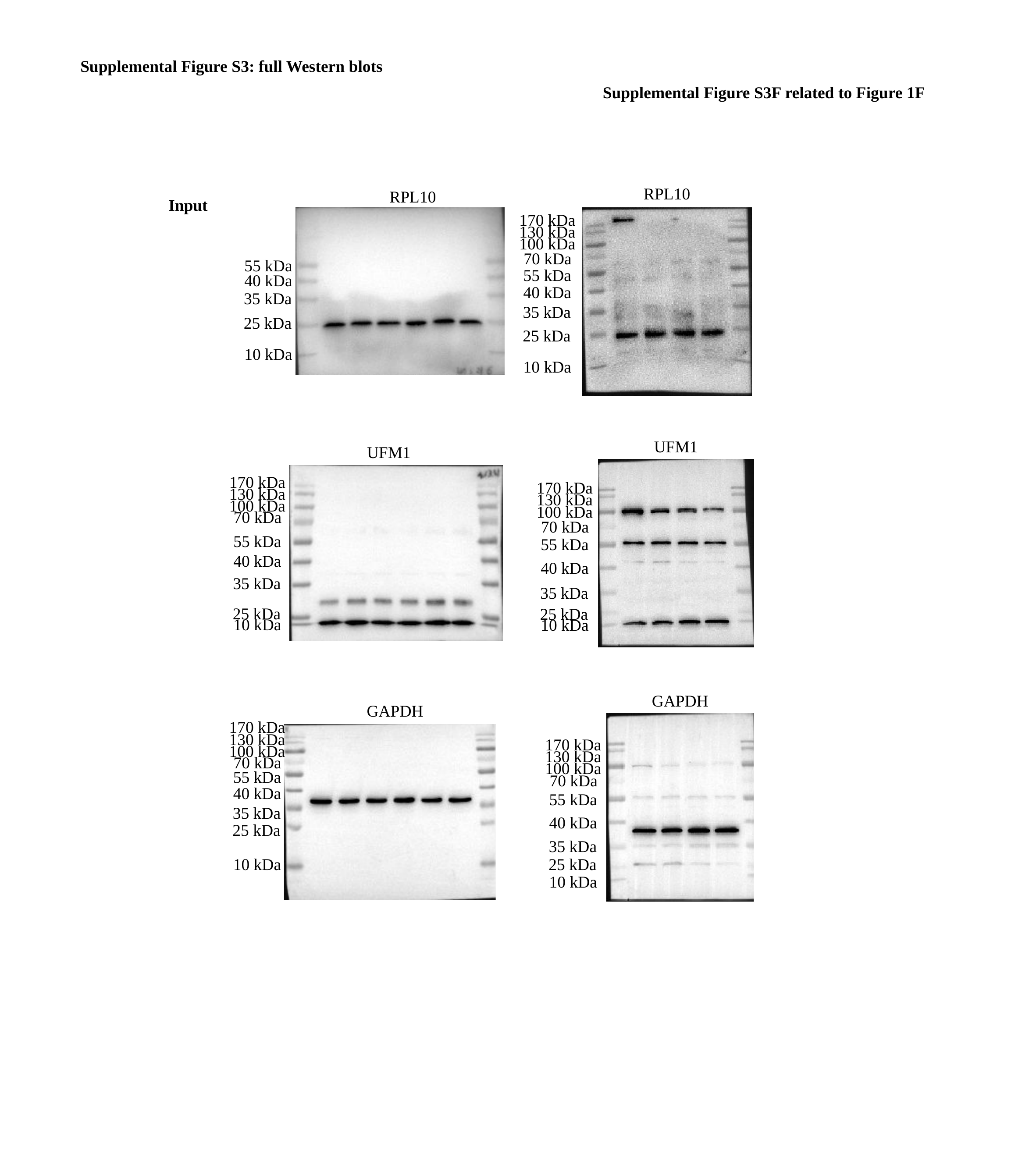

Supplemental Figure S3: full Western blots
Supplemental Figure S3F related to Figure 1F
RPL10
RPL10
 Input
170 kDa
130 kDa
100 kDa
70 kDa
55 kDa
55 kDa
40 kDa
40 kDa
35 kDa
35 kDa
25 kDa
25 kDa
10 kDa
10 kDa
UFM1
UFM1
170 kDa
170 kDa
130 kDa
130 kDa
100 kDa
100 kDa
70 kDa
70 kDa
55 kDa
55 kDa
40 kDa
40 kDa
35 kDa
35 kDa
25 kDa
25 kDa
10 kDa
10 kDa
GAPDH
GAPDH
170 kDa
130 kDa
170 kDa
100 kDa
130 kDa
70 kDa
100 kDa
55 kDa
70 kDa
40 kDa
55 kDa
35 kDa
40 kDa
25 kDa
35 kDa
25 kDa
10 kDa
10 kDa

## Slide 11
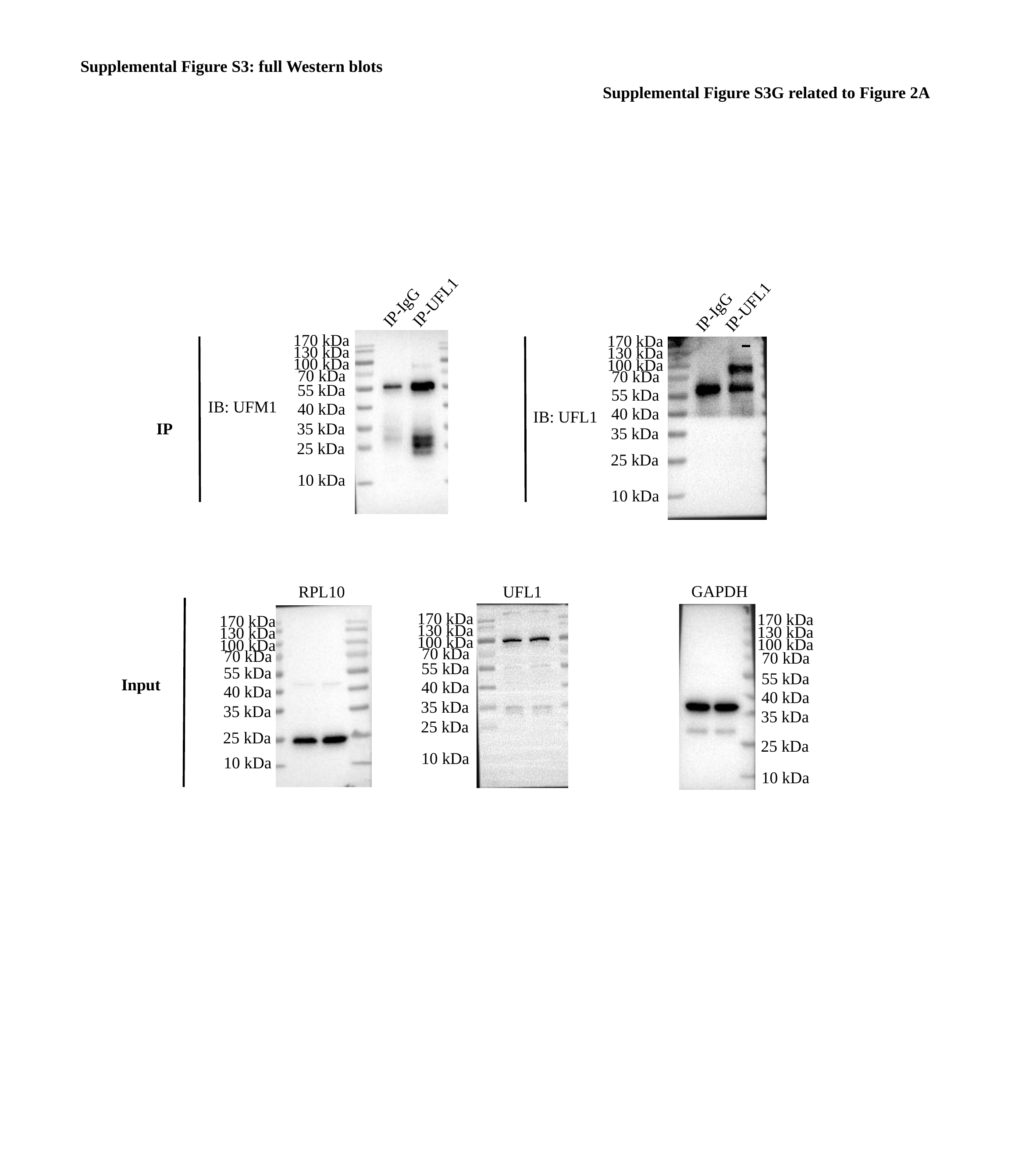

Supplemental Figure S3: full Western blots
Supplemental Figure S3G related to Figure 2A
IP-IgG
IP-UFL1
IP-IgG
IP-UFL1
170 kDa
170 kDa
130 kDa
130 kDa
100 kDa
100 kDa
70 kDa
70 kDa
55 kDa
55 kDa
IB: UFM1
40 kDa
40 kDa
IB: UFL1
35 kDa
IP
35 kDa
25 kDa
25 kDa
10 kDa
10 kDa
GAPDH
UFL1
RPL10
170 kDa
170 kDa
170 kDa
130 kDa
130 kDa
130 kDa
100 kDa
100 kDa
100 kDa
70 kDa
70 kDa
70 kDa
55 kDa
55 kDa
55 kDa
Input
40 kDa
40 kDa
40 kDa
35 kDa
35 kDa
35 kDa
25 kDa
25 kDa
25 kDa
10 kDa
10 kDa
10 kDa

## Slide 12
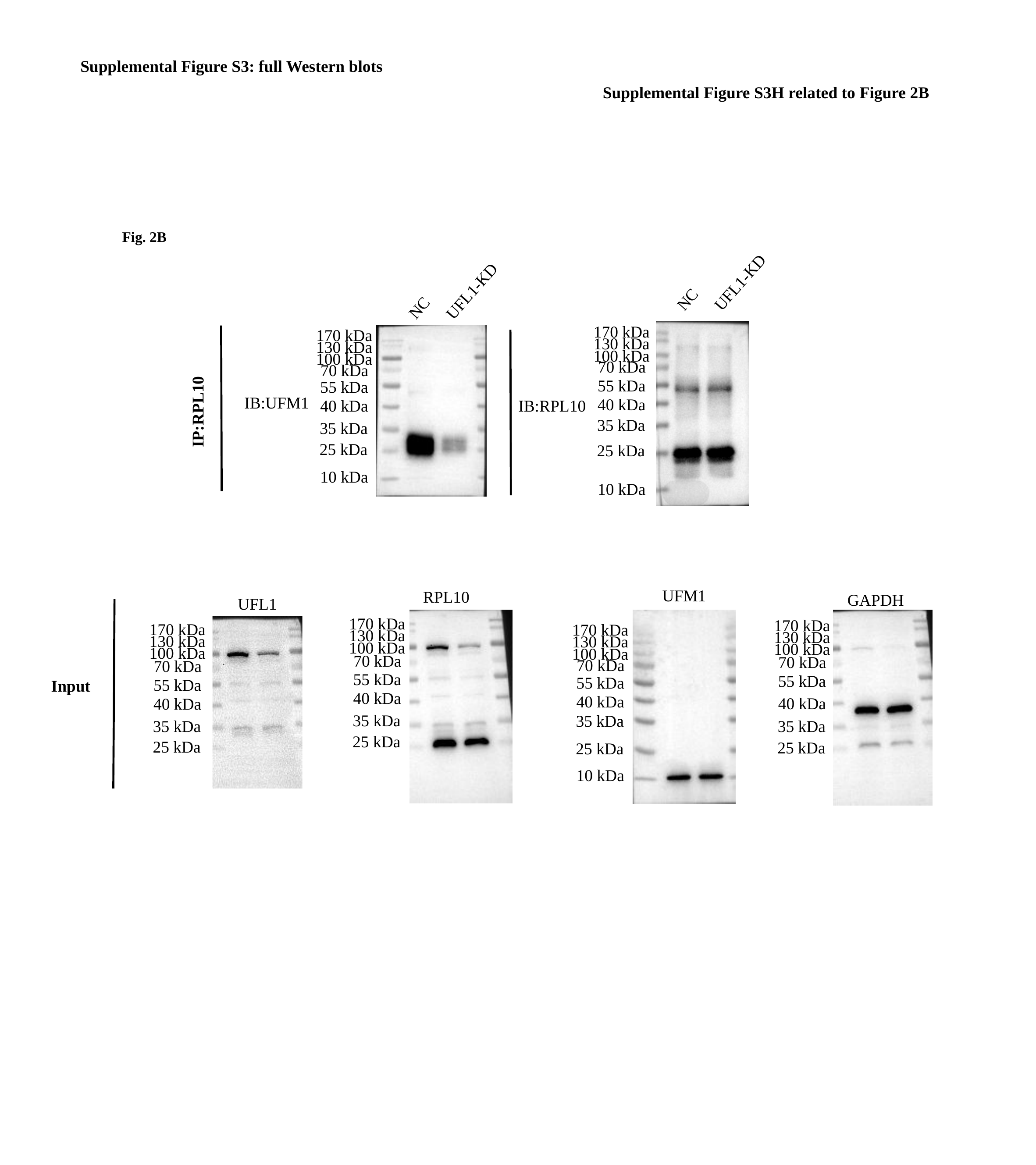

Supplemental Figure S3: full Western blots
Supplemental Figure S3H related to Figure 2B
Fig. 2B
UFL1-KD
NC
UFL1-KD
NC
170 kDa
170 kDa
130 kDa
130 kDa
100 kDa
100 kDa
70 kDa
70 kDa
55 kDa
55 kDa
IB:UFM1
40 kDa
40 kDa
IB:RPL10
IP:RPL10
35 kDa
35 kDa
25 kDa
25 kDa
10 kDa
10 kDa
UFM1
RPL10
GAPDH
UFL1
170 kDa
170 kDa
170 kDa
170 kDa
130 kDa
130 kDa
130 kDa
130 kDa
100 kDa
100 kDa
100 kDa
100 kDa
70 kDa
70 kDa
70 kDa
70 kDa
55 kDa
55 kDa
55 kDa
55 kDa
Input
40 kDa
40 kDa
40 kDa
40 kDa
35 kDa
35 kDa
35 kDa
35 kDa
25 kDa
25 kDa
25 kDa
25 kDa
10 kDa

## Slide 13
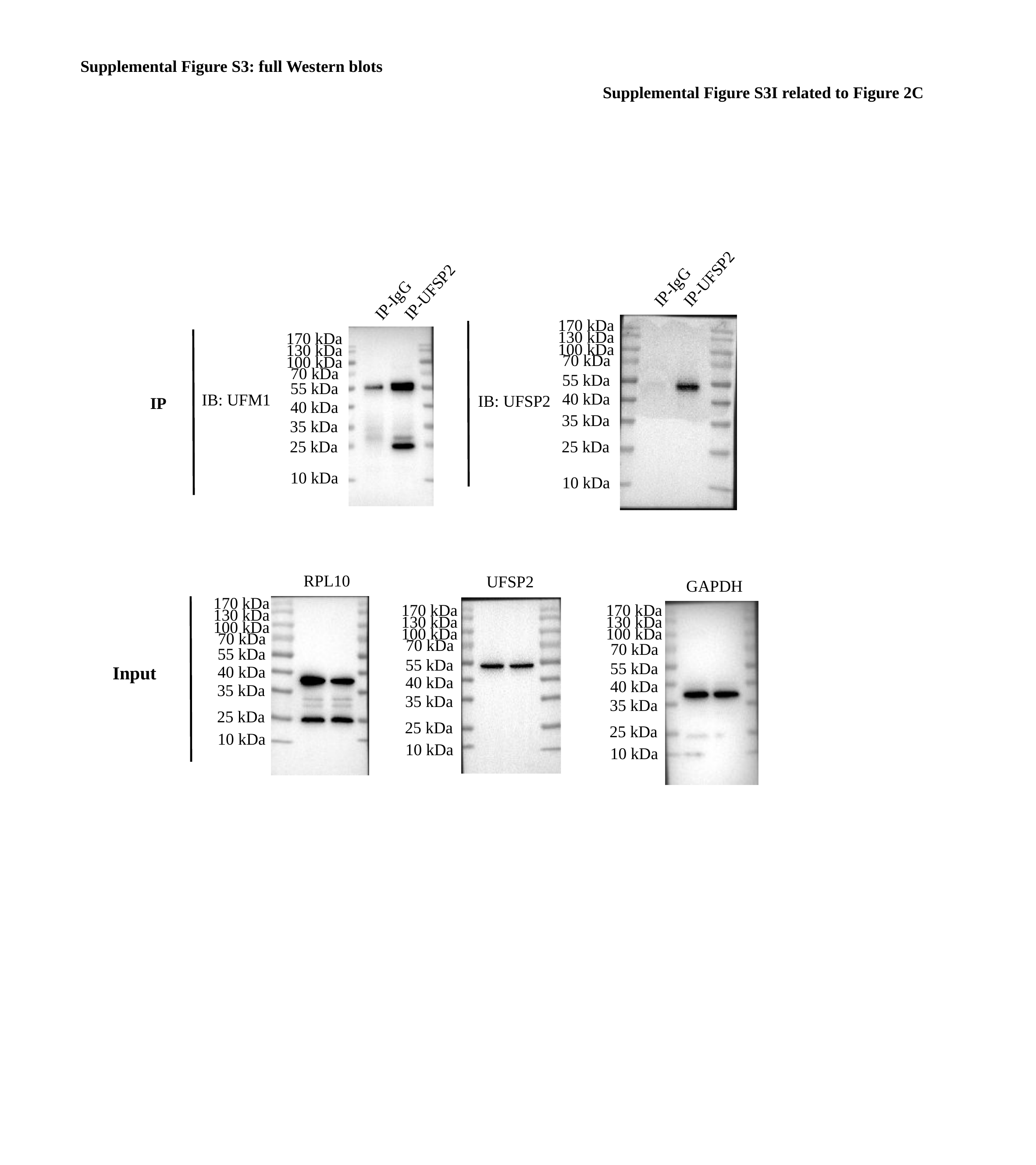

Supplemental Figure S3: full Western blots
Supplemental Figure S3I related to Figure 2C
IP-IgG
IP-UFSP2
IP-IgG
IP-UFSP2
170 kDa
130 kDa
170 kDa
100 kDa
130 kDa
70 kDa
100 kDa
70 kDa
55 kDa
55 kDa
40 kDa
IB: UFM1
IB: UFSP2
IP
40 kDa
35 kDa
35 kDa
25 kDa
25 kDa
10 kDa
10 kDa
RPL10
UFSP2
GAPDH
170 kDa
170 kDa
170 kDa
130 kDa
130 kDa
130 kDa
100 kDa
100 kDa
100 kDa
70 kDa
70 kDa
70 kDa
55 kDa
55 kDa
55 kDa
40 kDa
Input
40 kDa
40 kDa
35 kDa
35 kDa
35 kDa
25 kDa
25 kDa
25 kDa
10 kDa
10 kDa
10 kDa

## Slide 14
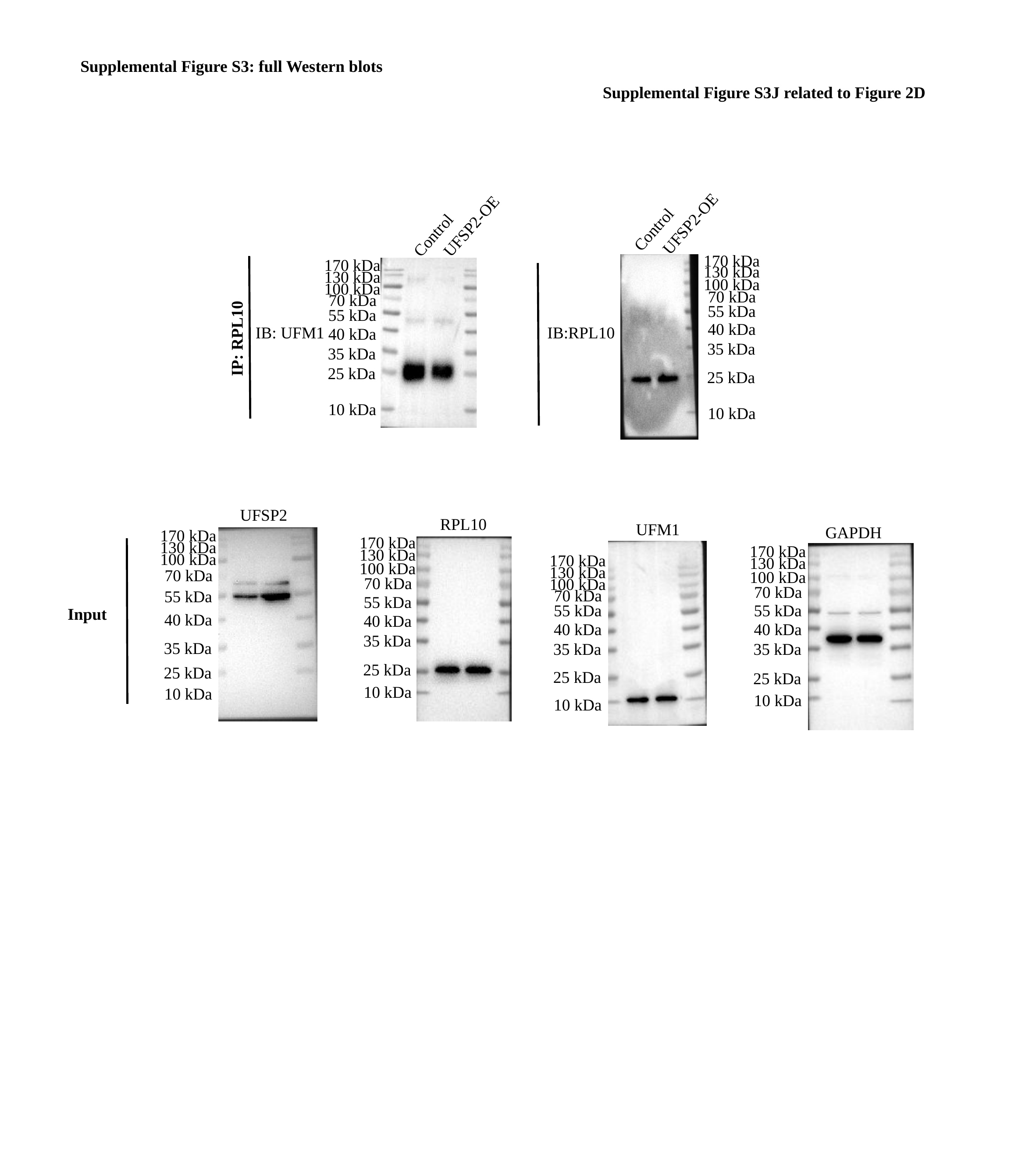

Supplemental Figure S3: full Western blots
Supplemental Figure S3J related to Figure 2D
UFSP2-OE
UFSP2-OE
Control
Control
170 kDa
170 kDa
130 kDa
130 kDa
100 kDa
100 kDa
70 kDa
70 kDa
55 kDa
55 kDa
40 kDa
IB:RPL10
IB: UFM1
40 kDa
IP: RPL10
35 kDa
35 kDa
25 kDa
25 kDa
10 kDa
10 kDa
UFSP2
RPL10
UFM1
GAPDH
170 kDa
170 kDa
130 kDa
170 kDa
130 kDa
100 kDa
170 kDa
130 kDa
100 kDa
130 kDa
70 kDa
100 kDa
70 kDa
100 kDa
70 kDa
70 kDa
55 kDa
55 kDa
55 kDa
55 kDa
Input
40 kDa
40 kDa
40 kDa
40 kDa
35 kDa
35 kDa
35 kDa
35 kDa
25 kDa
25 kDa
25 kDa
25 kDa
10 kDa
10 kDa
10 kDa
10 kDa

## Slide 15
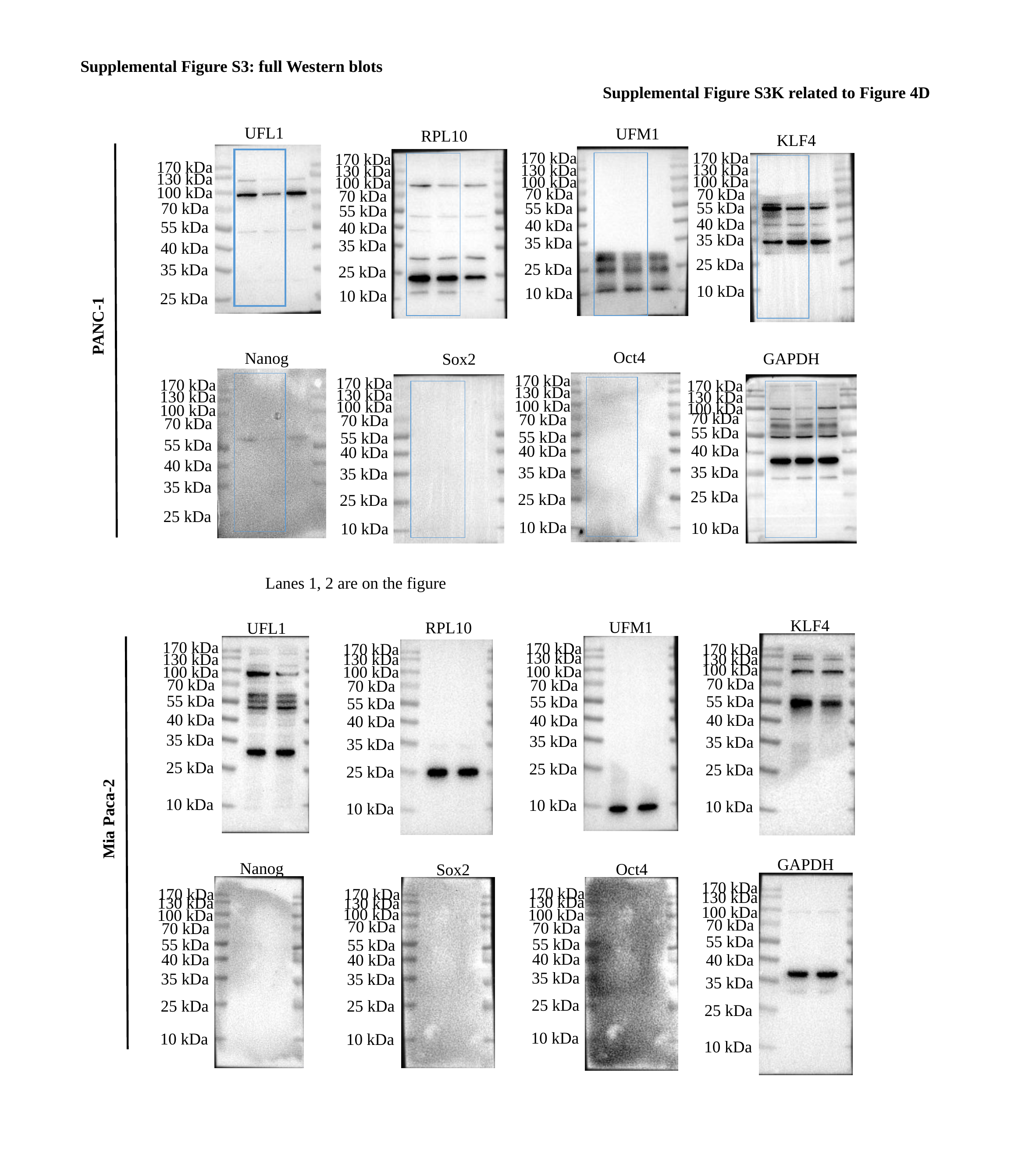

Supplemental Figure S3: full Western blots
Supplemental Figure S3K related to Figure 4D
UFL1
UFM1
RPL10
KLF4
170 kDa
170 kDa
170 kDa
170 kDa
130 kDa
130 kDa
130 kDa
130 kDa
100 kDa
100 kDa
100 kDa
100 kDa
70 kDa
70 kDa
70 kDa
55 kDa
55 kDa
70 kDa
55 kDa
40 kDa
40 kDa
55 kDa
40 kDa
35 kDa
35 kDa
35 kDa
40 kDa
25 kDa
25 kDa
35 kDa
25 kDa
10 kDa
10 kDa
10 kDa
25 kDa
PANC-1
Oct4
Nanog
GAPDH
Sox2
170 kDa
170 kDa
170 kDa
170 kDa
130 kDa
130 kDa
130 kDa
130 kDa
100 kDa
100 kDa
100 kDa
100 kDa
70 kDa
70 kDa
70 kDa
70 kDa
55 kDa
55 kDa
55 kDa
55 kDa
40 kDa
40 kDa
40 kDa
40 kDa
35 kDa
35 kDa
35 kDa
35 kDa
25 kDa
25 kDa
25 kDa
25 kDa
10 kDa
10 kDa
10 kDa
Lanes 1, 2 are on the figure
KLF4
UFM1
RPL10
UFL1
170 kDa
170 kDa
170 kDa
170 kDa
130 kDa
130 kDa
130 kDa
130 kDa
100 kDa
100 kDa
100 kDa
100 kDa
70 kDa
70 kDa
70 kDa
70 kDa
55 kDa
55 kDa
55 kDa
55 kDa
40 kDa
40 kDa
40 kDa
40 kDa
35 kDa
35 kDa
35 kDa
35 kDa
25 kDa
25 kDa
25 kDa
25 kDa
10 kDa
10 kDa
10 kDa
10 kDa
Mia Paca-2
GAPDH
Nanog
Oct4
Sox2
170 kDa
170 kDa
170 kDa
170 kDa
130 kDa
130 kDa
130 kDa
130 kDa
100 kDa
100 kDa
100 kDa
100 kDa
70 kDa
70 kDa
70 kDa
70 kDa
55 kDa
55 kDa
55 kDa
55 kDa
40 kDa
40 kDa
40 kDa
40 kDa
35 kDa
35 kDa
35 kDa
35 kDa
25 kDa
25 kDa
25 kDa
25 kDa
10 kDa
10 kDa
10 kDa
10 kDa

## Slide 16
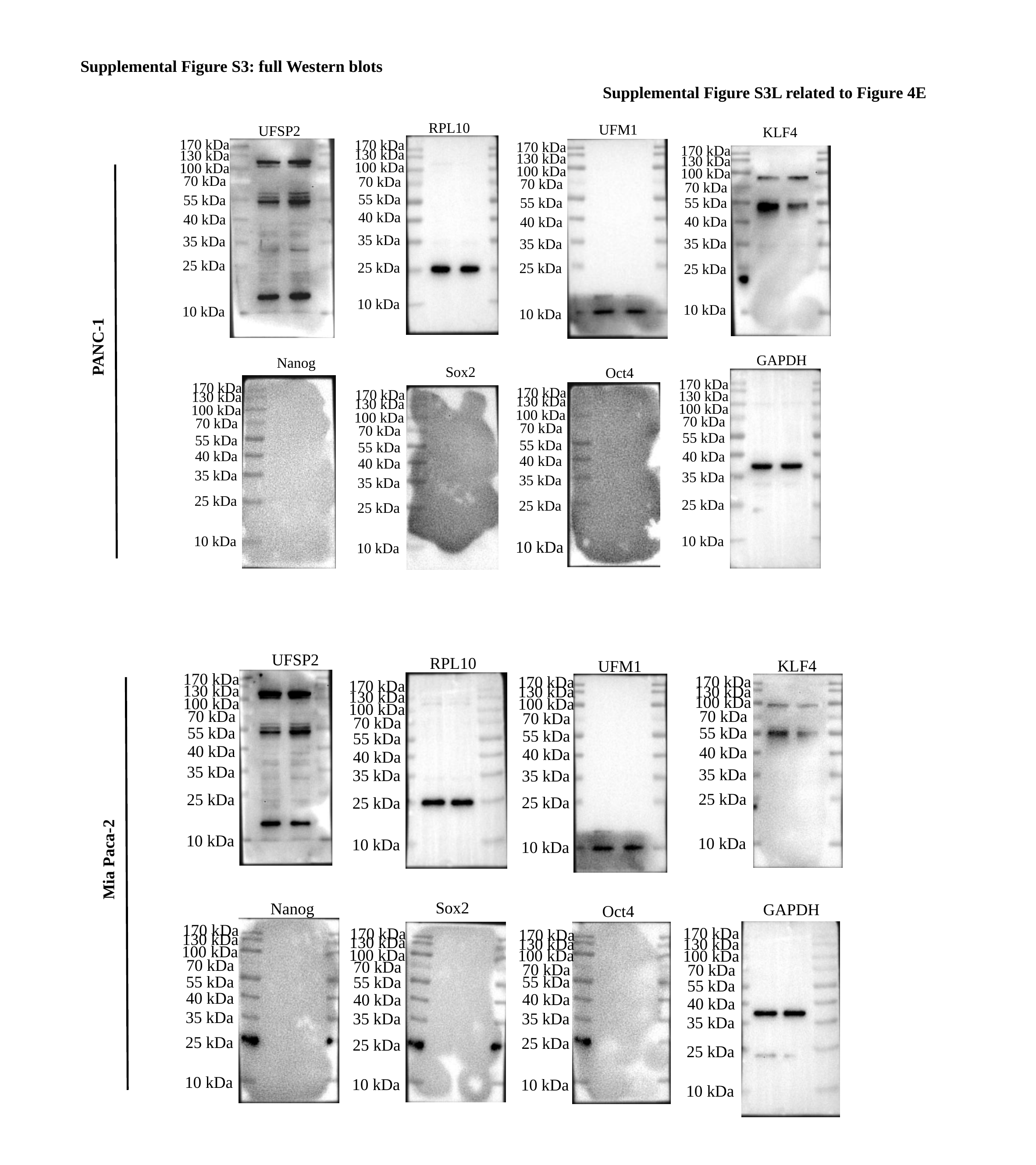

Supplemental Figure S3: full Western blots
Supplemental Figure S3L related to Figure 4E
RPL10
UFM1
UFSP2
KLF4
170 kDa
170 kDa
170 kDa
170 kDa
130 kDa
130 kDa
130 kDa
130 kDa
100 kDa
100 kDa
100 kDa
100 kDa
70 kDa
70 kDa
70 kDa
70 kDa
55 kDa
55 kDa
55 kDa
55 kDa
40 kDa
40 kDa
40 kDa
40 kDa
35 kDa
35 kDa
35 kDa
35 kDa
25 kDa
25 kDa
25 kDa
25 kDa
10 kDa
10 kDa
10 kDa
10 kDa
PANC-1
GAPDH
Nanog
Sox2
Oct4
170 kDa
170 kDa
170 kDa
170 kDa
130 kDa
130 kDa
130 kDa
130 kDa
100 kDa
100 kDa
100 kDa
100 kDa
70 kDa
70 kDa
70 kDa
70 kDa
55 kDa
55 kDa
55 kDa
55 kDa
40 kDa
40 kDa
40 kDa
40 kDa
35 kDa
35 kDa
35 kDa
35 kDa
25 kDa
25 kDa
25 kDa
25 kDa
10 kDa
10 kDa
10 kDa
10 kDa
UFSP2
RPL10
KLF4
UFM1
170 kDa
170 kDa
170 kDa
170 kDa
130 kDa
130 kDa
130 kDa
130 kDa
100 kDa
100 kDa
100 kDa
100 kDa
70 kDa
70 kDa
70 kDa
70 kDa
55 kDa
55 kDa
55 kDa
55 kDa
40 kDa
40 kDa
40 kDa
40 kDa
35 kDa
35 kDa
35 kDa
35 kDa
25 kDa
25 kDa
25 kDa
25 kDa
10 kDa
10 kDa
10 kDa
10 kDa
Mia Paca-2
Sox2
Nanog
GAPDH
Oct4
170 kDa
170 kDa
170 kDa
170 kDa
130 kDa
130 kDa
130 kDa
130 kDa
100 kDa
100 kDa
100 kDa
100 kDa
70 kDa
70 kDa
70 kDa
70 kDa
55 kDa
55 kDa
55 kDa
55 kDa
40 kDa
40 kDa
40 kDa
40 kDa
35 kDa
35 kDa
35 kDa
35 kDa
25 kDa
25 kDa
25 kDa
25 kDa
10 kDa
10 kDa
10 kDa
10 kDa

## Slide 17
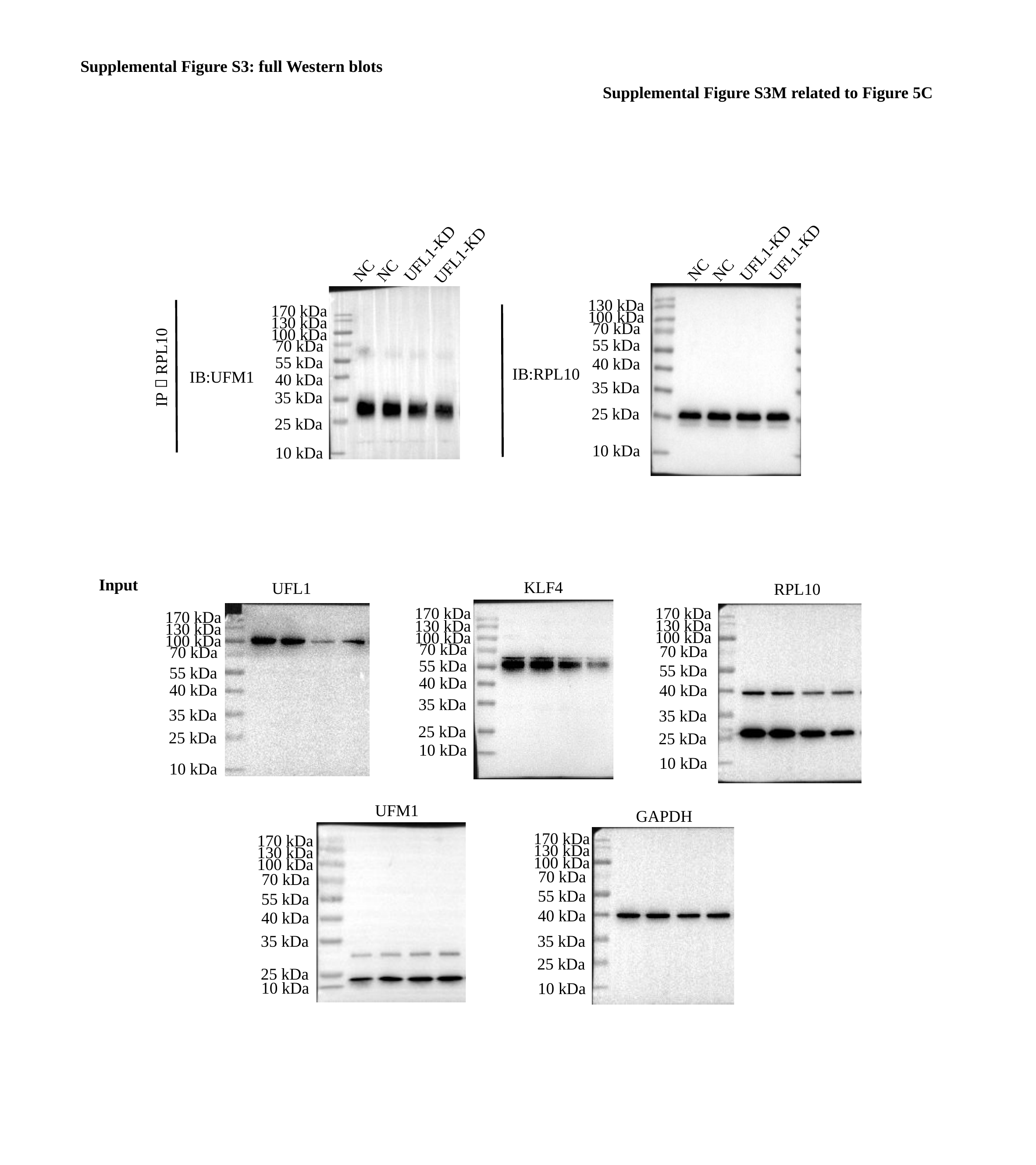

Supplemental Figure S3: full Western blots
Supplemental Figure S3M related to Figure 5C
UFL1-KD
UFL1-KD
UFL1-KD
UFL1-KD
NC
NC
NC
NC
130 kDa
170 kDa
100 kDa
130 kDa
70 kDa
100 kDa
55 kDa
70 kDa
55 kDa
40 kDa
IP：RPL10
IB:RPL10
IB:UFM1
40 kDa
35 kDa
35 kDa
25 kDa
25 kDa
10 kDa
10 kDa
Input
KLF4
UFL1
RPL10
170 kDa
170 kDa
170 kDa
130 kDa
130 kDa
130 kDa
100 kDa
100 kDa
100 kDa
70 kDa
70 kDa
70 kDa
55 kDa
55 kDa
55 kDa
40 kDa
40 kDa
40 kDa
35 kDa
35 kDa
35 kDa
25 kDa
25 kDa
25 kDa
10 kDa
10 kDa
10 kDa
UFM1
GAPDH
170 kDa
170 kDa
130 kDa
130 kDa
100 kDa
100 kDa
70 kDa
70 kDa
55 kDa
55 kDa
40 kDa
40 kDa
35 kDa
35 kDa
25 kDa
25 kDa
10 kDa
10 kDa

## Slide 18
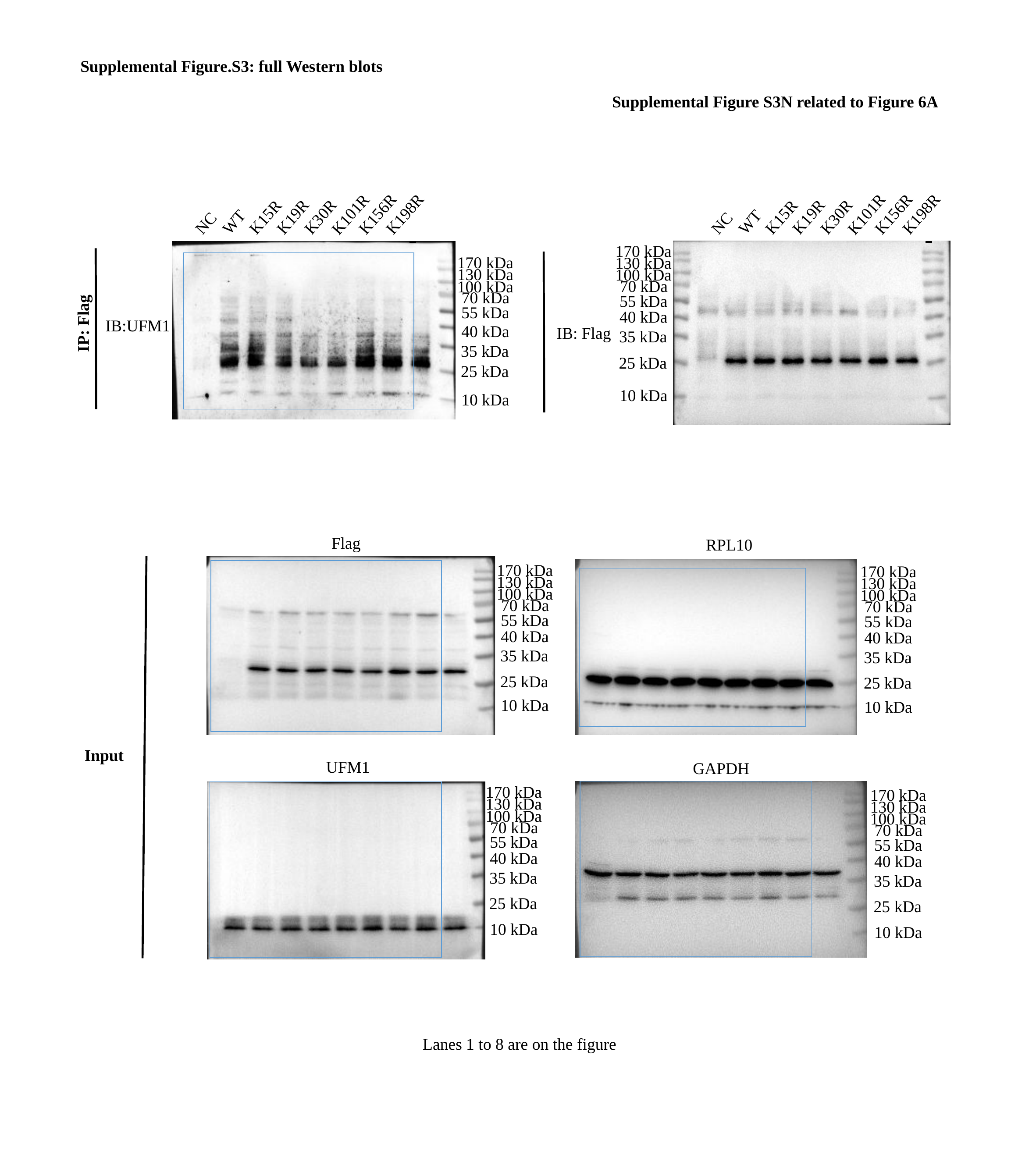

Supplemental Figure.S3: full Western blots
Supplemental Figure S3N related to Figure 6A
K101R
K101R
K30R
K30R
K156R
K156R
K19R
K19R
K15R
K15R
K198R
K198R
NC
NC
WT
WT
170 kDa
170 kDa
130 kDa
130 kDa
100 kDa
70 kDa
100 kDa
70 kDa
55 kDa
55 kDa
40 kDa
IP: Flag
IB:UFM1
40 kDa
IB: Flag
35 kDa
35 kDa
25 kDa
25 kDa
10 kDa
10 kDa
Flag
RPL10
170 kDa
170 kDa
130 kDa
130 kDa
100 kDa
100 kDa
70 kDa
70 kDa
55 kDa
55 kDa
40 kDa
40 kDa
35 kDa
35 kDa
25 kDa
25 kDa
10 kDa
10 kDa
Input
UFM1
GAPDH
170 kDa
170 kDa
130 kDa
130 kDa
100 kDa
100 kDa
70 kDa
70 kDa
55 kDa
55 kDa
40 kDa
40 kDa
35 kDa
35 kDa
25 kDa
25 kDa
10 kDa
10 kDa
Lanes 1 to 8 are on the figure

## Slide 19
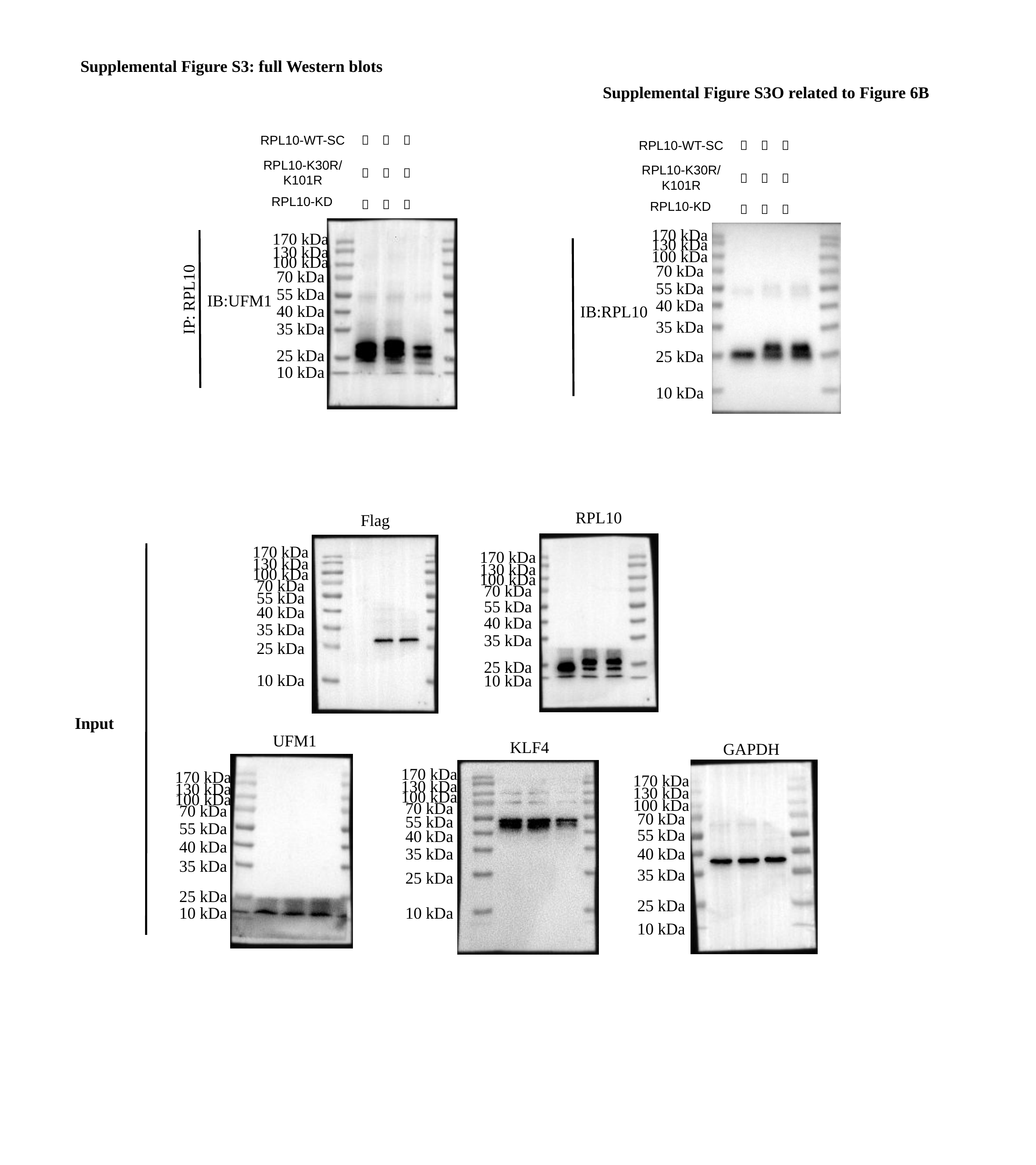

Supplemental Figure S3: full Western blots
Supplemental Figure S3O related to Figure 6B
RPL10-WT-SC
－ ＋ －
RPL10-K30R/K101R
－ － ＋
RPL10-KD
－ ＋ ＋
RPL10-WT-SC
－ ＋ －
RPL10-K30R/K101R
－ － ＋
RPL10-KD
－ ＋ ＋
170 kDa
170 kDa
130 kDa
130 kDa
100 kDa
100 kDa
70 kDa
70 kDa
55 kDa
55 kDa
IP: RPL10
IB:UFM1
40 kDa
40 kDa
IB:RPL10
35 kDa
35 kDa
25 kDa
25 kDa
10 kDa
10 kDa
RPL10
Flag
170 kDa
170 kDa
130 kDa
130 kDa
100 kDa
100 kDa
70 kDa
70 kDa
55 kDa
55 kDa
40 kDa
40 kDa
35 kDa
35 kDa
25 kDa
25 kDa
10 kDa
10 kDa
 Input
UFM1
KLF4
GAPDH
170 kDa
170 kDa
170 kDa
130 kDa
130 kDa
130 kDa
100 kDa
100 kDa
100 kDa
70 kDa
70 kDa
70 kDa
55 kDa
55 kDa
55 kDa
40 kDa
40 kDa
40 kDa
35 kDa
35 kDa
35 kDa
25 kDa
25 kDa
25 kDa
10 kDa
10 kDa
10 kDa

## Slide 20
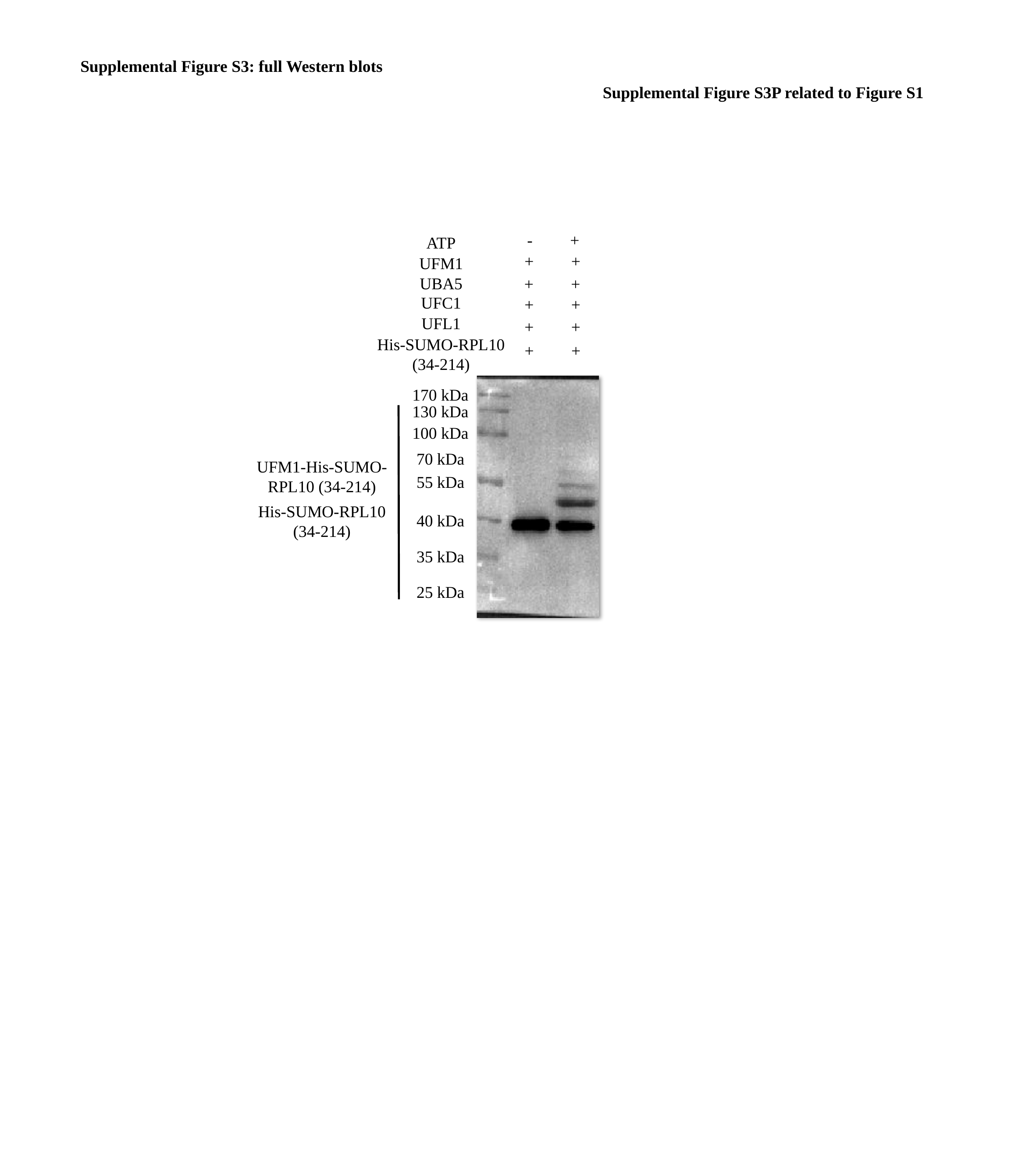

Supplemental Figure S3: full Western blots
Supplemental Figure S3P related to Figure S1
- +
ATP
+ +
UFM1
UBA5
+ +
UFC1
+ +
UFL1
+ +
His-SUMO-RPL10 (34-214)
+ +
170 kDa
130 kDa
100 kDa
70 kDa
UFM1-His-SUMO-RPL10 (34-214)
55 kDa
His-SUMO-RPL10 (34-214)
40 kDa
35 kDa
25 kDa
